# Heterogeneous conversion of CO_2_ into cyclic carbonates at ambient pressure catalyzed by ionothermal-derived meso-macroporous hierarchical poly(ionic liquid)s†
†Electronic supplementary information (ESI) available: Experimental section, details of the IR, ^13^C NMR and XPS spectra, characterization (^1^H NMR, ^13^C NMR, ^13^C CP-MAS NMR, TG, XRD, SEM, elemental analysis), N_2_ adsorption–desorption isotherms, pore size distribution, recycling test of the catalyst, comparison of the literature catalytic activity of different heterogeneous catalysts. See DOI: 10.1039/c5sc02050f


**DOI:** 10.1039/c5sc02050f

**Published:** 2015-08-27

**Authors:** Xiaochen Wang, Yu Zhou, Zengjing Guo, Guojian Chen, Jing Li, Yuming Shi, Yangqing Liu, Jun Wang

**Affiliations:** a State Key Laboratory of Materials-Oriented Chemical Engineering , College of Chemistry and Chemical Engineering , Nanjing Tech University (former Nanjing University of Technology) , Nanjing , Jiangsu 210009 , P. R. China . Email: junwang@njtech.edu.cn

## Abstract

Meso-macroporous hierarchical poly(ionic liquid)s (MPILs) with extremely high ionic site densities and tunable pore structures were ionothermally synthesized through the free radical self-polymerization of our newly designed rigid bis-vinylimidazolium salt monomer. The synthesis avoided the use of any templates, gave a high yield (>99%) and allowed recycling of the IL solvent; thus it is facile, atom-efficient, environmentally friendly and sustainable. The synthesized MPILs possessed distinctive features of polycation matrices, abundant halogen anions, and large surface areas. They not only presented enhanced CO_2_ capture, but led to breakthroughs in the heterogeneous catalytic conversion of CO_2_ into cyclic carbonates: (1) unprecedented high activity at atmospheric pressure and low temperature; (2) good substrate compatibility, even being active towards the extremely inert aliphatic long carbon-chain alkyl epoxides. This result renders the first occasion of a metal–solvent–additive free recyclable heterogeneous cycloaddition of CO_2_ at such mild conditions.

## Abstract 2

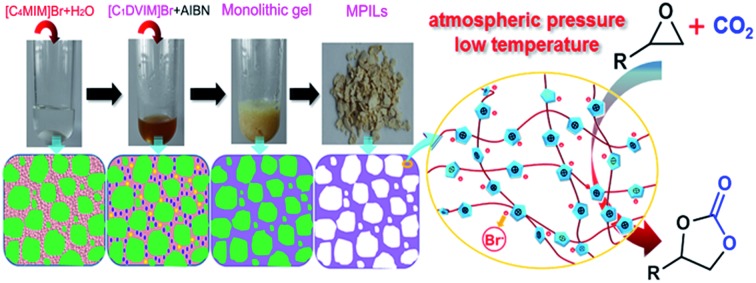
Meso-macroporous hierarchical poly(ionic liquid)s are ionothermally synthesized through self-polymerization of the new divinylimidazole IL, exhibiting enhanced CO_2_ capture and excellent activity in the cycloaddition of CO_2_ to epoxides.

## Introduction

Nanoporous polymeric materials have tremendously attracted continuous and increasing attention due to their versatile and tunable structures ranging from atomic/molecular to the nano/micrometer level and even the macroscopic scale.^1^–^3^ A great variety of functional nanoporous organic materials have been developed and have shown outstanding performances in numerous applications including gas storage, separation, catalysis, energy conversion, optoelectronics, bioengineering and so on.^4^,^5^ The task-specific design and fabrication of nanoporous polymers through facile, atomic efficient and sustainable strategies has become a hot topic in material science, energy and environment concerning fields, *etc.*^6^,^7^ Most of these existing nanoporous polymers have neutral skeletons. Ionic porous polymers with a high density of ionic sites may broaden the applications of polymeric materials; however, only in recent years has the preparation and application of ionic porous polymers attracted increasing research interests.^8^–^10^

The capture and utilization of carbon dioxide (CO_2_) is of particular importance for environmentally friendly processes and the sustainable development of human society, because it involves both the fight against the escalating levels of atmospheric global-warming gases and the efficient use of the most environmentally abundant available source of a C_1_ building block to produce high-value products.^11^–^13^ In this context, one challenge is to convert CO_2_ at mild conditions, *e.g.* atmospheric pressure and low temperature.^14^ Extensive efforts are being devoted to develop efficient adsorbents and catalysts.^15^–^19^ Among them, ionic liquids (ILs) or related materials have become a class of promising candidates for both CO_2_ capture and conversion.^20^–^23^ To promote separation and reusability of ILs, more attention is being paid to polymeric ILs (PILs), but they usually present inferior performance due to their limited porosity, which hampers mass transfer and the accessibility of the active sites.^24^ Alternatively, mesoporous PILs (MPILs) are more attractive as they combine the features of mesoporous materials, polymers and ILs.^25^–^28^ Nonetheless, it is still a great challenge to facilely prepare MPILs with a large surface area and a required task-specific framework composition.

In this work, we report a family of vinylimidazolium salt based meso-macroporous hierarchical MPIL monolithic materials through a facile, atom-efficient and sustainable pathway. The bis-vinylimidazolium salt of this work is not a conventional IL due to its high melting point (>100 °C), so the resultant MPILs here can also be called poly(imidazolium salt)s. The synthesis relies on the free radical self-polymerization of the bis-vinylimidazolium salt monomer by using another conventional IL as the solvent, namely ionothermal synthesis.

The principal advantages of this synthetic approach are the avoidance of an additional template and a volatile organic solvent, enabling the synthesis at ambient pressure.^29^ In addition, the IL solvent can be recycled to reduce the released waste, thus the route is safe, environmentally friendly and atom-efficient. An ionothermal route is applied in various syntheses for porous materials such as zeolites,^30^ metal–organic frameworks (MOFs),^31^ mesoporous inorganic salts,^32^ porous polymers,^33^*etc.*; however, to our knowledge, an ionothermal synthesis for producing large surfaced MPILs has never been achieved before. Herein, an ionothermal synthesis is successfully applied for the preparation of MPILs, producing large surfaced MPILs composed of polycations with abundant halogen anions. Halogen anions in ILs have been revealed to be active in CO_2_ conversion.^34^–^36^ It is thus rational to expect that a nanoporous polymer with a large surface area and a halogen anion-enriched skeleton should be highly efficient for heterogeneous CO_2_ capture and conversion. Indeed, application assessments prove that the present ionothermally synthesized nanoporous polymer exhibits enhanced CO_2_ capture and excellent performance in the cycloaddition of CO_2_ to epoxides, with facile and stable recycling, good substrate compatibility even towards long carbon-chain alkyl epoxides, and a high activity at atmospheric pressure and low temperature.

## Results and discussion

**Scheme 1:**
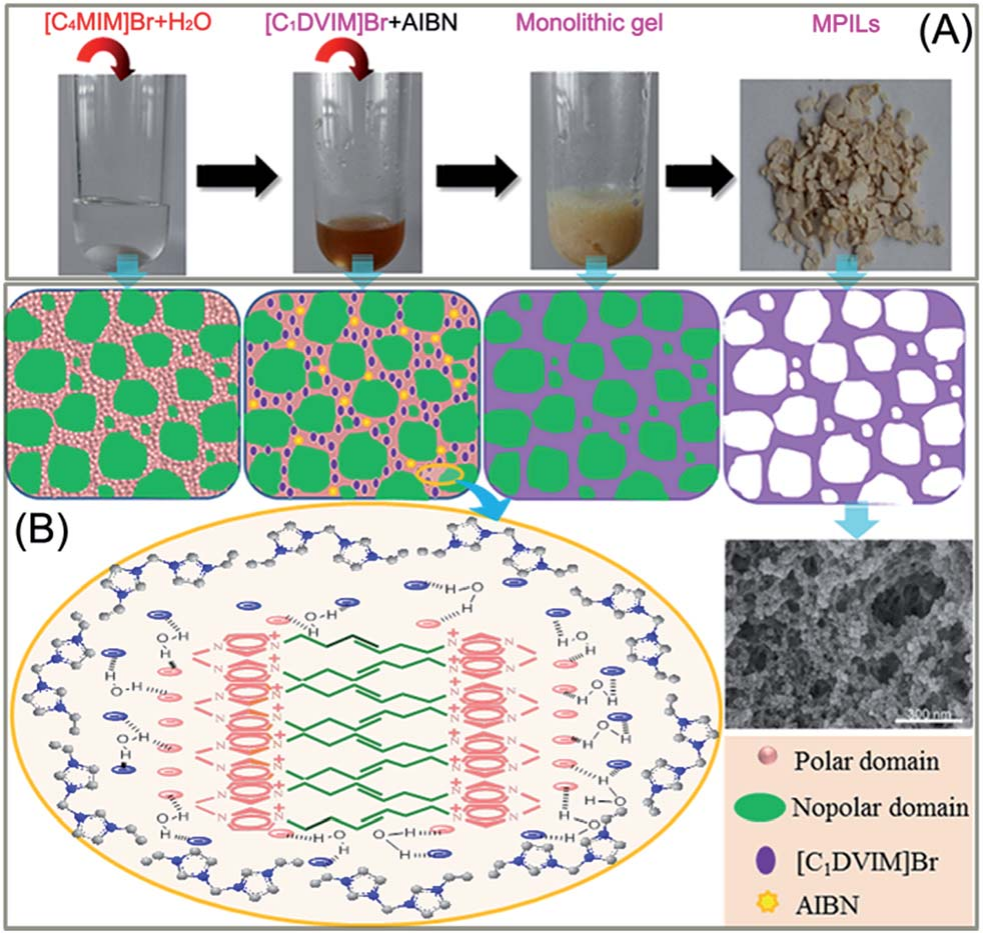
(A) Photographs of the ionothermal synthesis of a mesoporous poly(ionic liquid); (B) schematic illustration of the possible mechanism for the formation of the mesoporous structure.

**Scheme 2:**
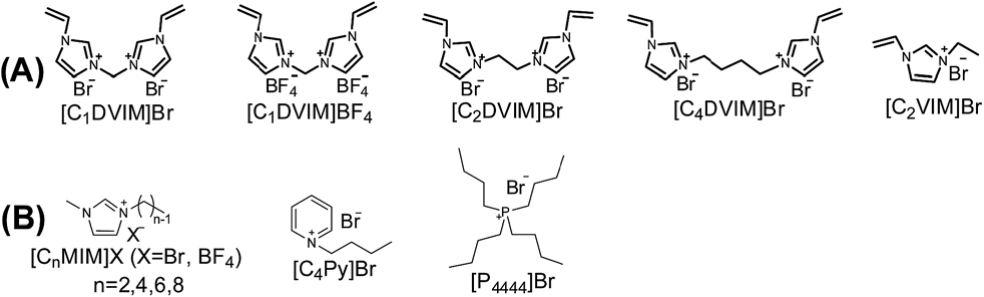
(A) Structure of the synthesized vinylimidazolium salt monomers and (B) structures of the IL solvents.

**Fig. 1:**
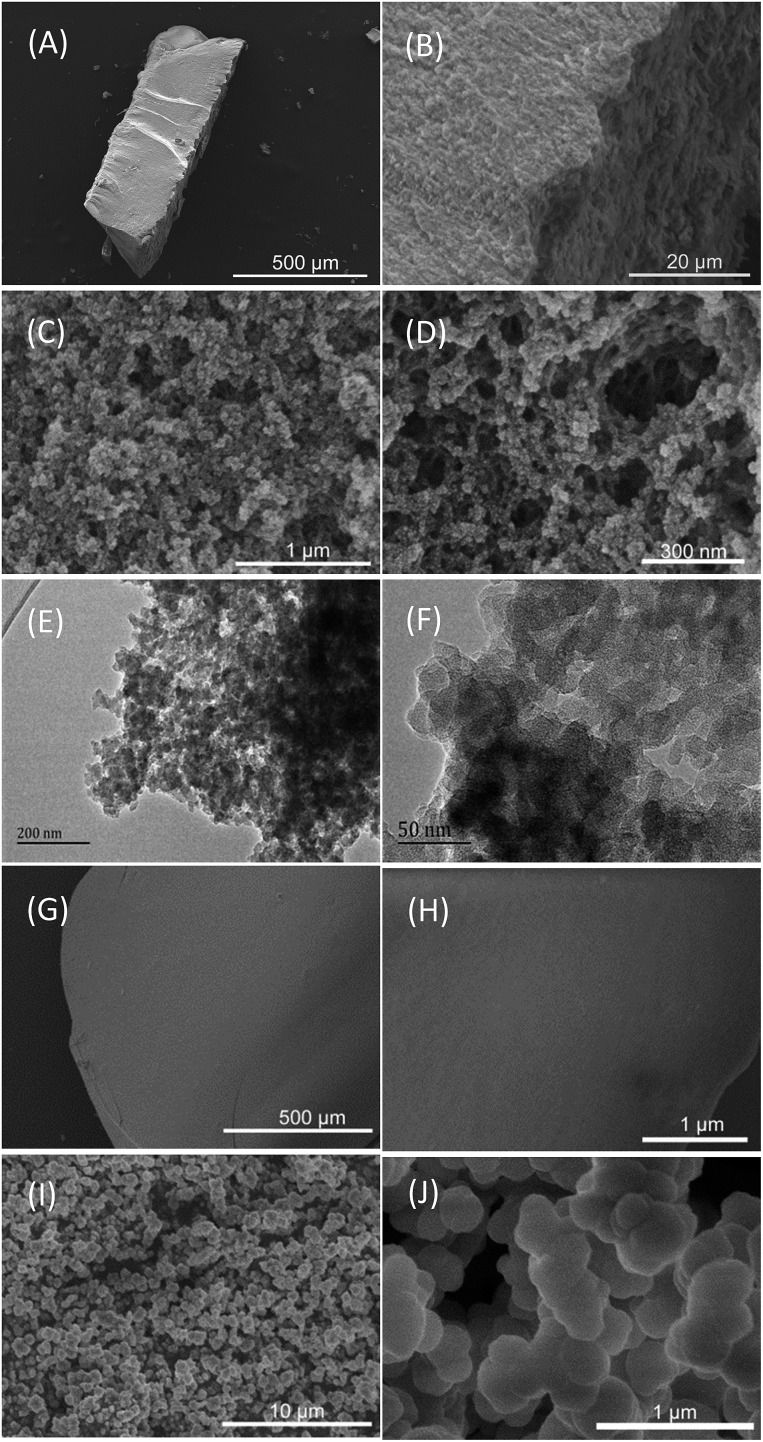
(A–D) SEM and (E and F) TEM images of PDMBr. SEM images of (G and H) PDMBr-H and (I and J) PDMBr-E.

**Fig. 2:**
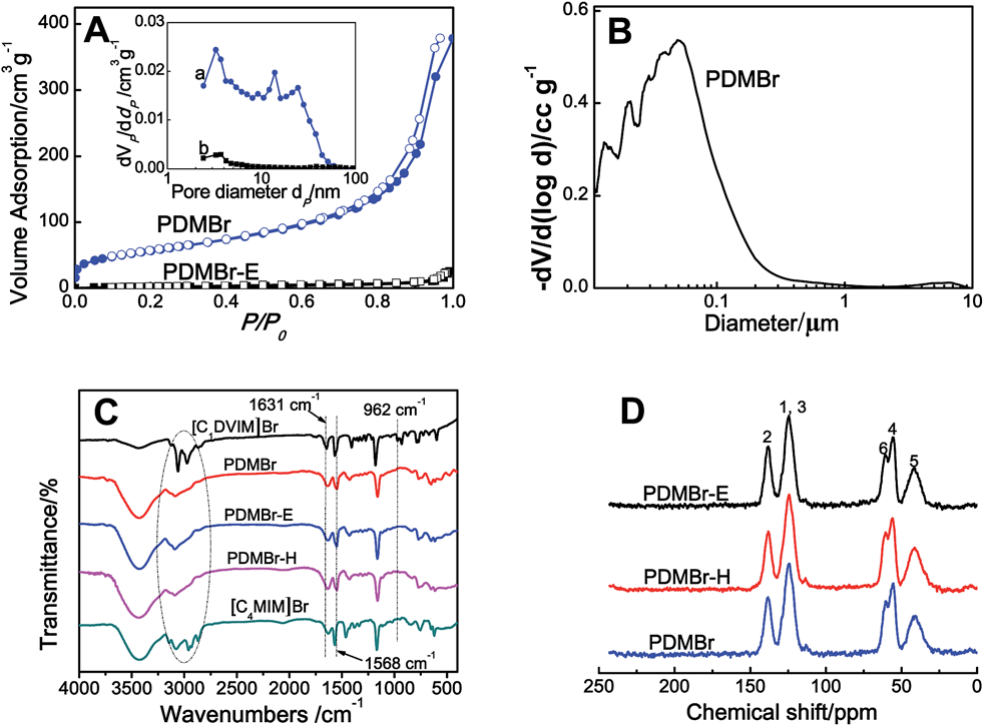
(A) N_2_ sorption isotherm and pore size distribution curve (inset); (B) macropore size distribution, detected using mercury inclusion porosimetry; (C) FT-IR and (D) ^13^C MAS NMR spectra of the different samples.

Scheme 1A shows the synthetic procedure, involving the dissolution of the vinylimidazolium salt monomer and initiator AIBN (azodiisobutyronitrile) in the IL solvent (a small amount of water is added as a co-solvent when needed), followed by self-polymerization at 100 °C for 24 h. Various imidazolium salt monomers and different IL solvents are studied, seen in Scheme 2A and B. Monolithic MPILs are obtained from a bis-vinylimidazolium salt monomer, typically [C_1_DVIM]Br with only one carbon bridging two vinylimidazolium rings, that forms a rigid structure. Herein, [C_1_DVIM]Br is the new imidazolium salt compound that we specifically designed, prepared and characterized (Fig. S1†), of which the lead MPIL sample PDMBr is synthesized with the initial composition of 0.3 g [C_1_DVIM]Br, 0.03 g AIBN, 6 g [C_4_MIM]Br (IL solvent) and 0.75 g H_2_O. The synthetic solution is solidified after polymerization, producing a monolithic gel. After washing and drying, a rigid monolith at the millimeter or centimeter level is achieved (Scheme 1A). SEM images show an apparent hierarchical meso-macroporous structure (Fig. 1A–D). The primary particles are at the nanoscale with widths of around 20 nm and lengths ranging tens of nanometers. They are interconnected with each other to form a cross-linked framework with pore sizes ranging from several to tens of nanometers, which is further confirmed by the TEM images (Fig. 1E and F). The nitrogen sorption analysis displays a type IV isotherm for PDMBr, indicative of a classical mesoporous structure (Fig. 2A). The surface area and pore volume are 205 m^2^ g^–1^ and 0.57 cm^3^ g^–1^ (Table 1, entry 2), respectively. The macropore size distribution, detected using mercury inclusion porosimetry, indicates that the porosity degree of PDMBr is 44.6% (Fig. 2B), giving macropore sizes ranging from the nano to micrometer scale. The above analyses demonstrate the formation of a meso-macroporous hierarchical monolithic structure.

**Table 1:** Textural properties and catalytic performance of the different samples^*a*^

Entry	Catalyst	*S*_BET_ ^*b*^ (m^2^ g^–1^)	*V*_p_ ^*c*^ (cm^3^ g^–1^)	*D*_av_ ^*d*^ (nm)	Con.^*e*^ (%)	Sel.^*f*^ (%)
1	[C_1_DVIM]Br	—	—	—	98.8	98.9
2	PDMBr	205	0.57	11.0	99.0	98.5
3	PDMBr-E	7.0	0.04	22.9	61.8	95.0
4^*g*^	PDMBr-H	—	—	—	28.0	17.8
5^*h*^	PDMBF	224	0.40	7.2	35.1	61.8

^*a*^Reaction conditions: SO 10 mmol, catalyst 0.05 g (1.3 mol%), CO_2_ 1.0 MPa, 110 °C, 4 h.

^*b*^BET surface area.

^*c*^Total pore volume.

^*d*^Average pore diameter.

^*e*^Determined using GC with *n*-dodecane as an internal standard.

^*f*^Phenylacetaldehyde was identified as the only by-product.

^*g*^Nonporous.

^*h*^Prepared similarly to PDMBr except that [C_1_DVIM]BF_4_ was used as the monomer.

For further characterization, the FT-IR (Fig. 2C), XPS (Fig. S4†) and ^13^C MAS NMR spectra (Fig. 2D) of PDMBr also revealed the successful polymerization of the bis-vinylimidazolium salt. Moreover, the elemental analysis (Table S2†), ^13^C NMR and FT-IR spectra of PDMBr are the same as those of PDMBr-E and PDMBr-H, indicating the same framework composition of these samples and the successful removal of IL solvent. The details for the above explanations are described in the ESI.† The TG analysis shows that PDMBr has satisfactory thermal stability with decomposition starting at 260 °C (Fig. S5†), which is similar to the stability of PDMBr-H but higher than that of PDMBr-E (220 °C). The XRD patterns show the amorphous texture of these polymers (Fig. S6†). These analyses reveal that the polymerization in the IL solvent is similar to that from the hydrothermal or solvothermal routes, except for the porogenic effect.

After polymerization, the IL solvent [C_4_MIM]Br can be almost completely recovered with high purity, as verified by the ^1^H NMR analysis (Fig. S7†). The utilization of the recovered IL solvent in the synthesis gives a very similar pore structure to the initial PDMBr sample (Table S1,† entry 8), suggesting good reusability of the IL solvent. Thus, the synthesis is potentially a reduced-waste, atom-efficient and environmentally friendly sustainable pathway.

The influences of the polymerizable monomers and solvents were studied to further understand the pore formation. [C_2_DVIM]Br and [C_4_DVIM]Br with double-carbon and four-carbon chains, respectively, connecting two vinylimidazolium rings have been employed by Ghazali-Esfahani *et al.* for the preparation of nonporous polymeric ionic liquids.^34^ When the two monomers were used in this work, poor porosity with a much lowered surface area of 45 m^2^ g^–1^ was obtained from the less rigid monomer [C_2_DVIM]Br, and the more flexible monomer [C_4_DVIM]Br only yielded an absolutely nonporous structure (Fig. S8 and Table S3†). Also, the monocationic counterpart [C_2_VIM]Br gave rise to a nonporous product. Therefore, the specifically designed structurally rigid monomer [C_1_DVIM]Br is the prerequisite for creating abundant porosity. Several other IL solvents of [C_*n*_MIM]Br (*n* = 2, 6, 8) were surveyed (Fig. S9 and S11; Table S4†). Structural analyses indicated that the samples synthesized in [C_2_MIM]Br or [C_6_MIM]Br both possessed abundant porosity, comparable to PDMBr synthesized in [C_4_MIM]Br. The pore volume increases with the prolonged alkyl length in the IL solvent. However, the synthesis in [C_8_MIM]Br suffered a great volume shrinkage during the drying process, bringing about a nonporous structure, which implied collapse of the pre-formed meso-structure. Such a phenomenon suggests that the rigidity of the employed IL solvent is also important for the pore formation. In addition, other non-imidazolium IL media ([C_4_Py]Br and [P_4444_]Br) were tried in syntheses using the monomer [C_1_DVIM]Br, producing nonporosity or low surface areas (Fig. S10 and S11; Table S4†). The samples were also synthesized using ionic salts of TPABr (tetrapropyl ammonium bromide) and TBABr (tetrabutyl ammonium bromide). The results indicate that the highest surface area of 260 m^2^ g^–1^ is found for TPABr, and the largest pore volume of 0.92 cm^3^ g^–1^ is achieved for TBABr. The above results reflect the significant influence of the IL solvents on the pore generation.

Several other synthetic parameters are investigated to additionally adjust the composition and structure of MPILs. Varying the amount of IL solvent [C_4_MIM]Br and co-solvent H_2_O provides a series of MPILs with different morphologies and porosities (Fig. S12–14, Tables S1 and S5†), showing that suitable amounts of [C_4_MIM]Br and H_2_O account for the large surface area. Changing H_2_O to another organic solvent (ethanol, AcOH, DMF or DMSO) causes poor porosity, suggesting that a small amount of H_2_O favors pore formation (Fig. S15 and Table S6†). The anions of the obtained MPILs can be adjusted to be BF_4_^–^ by using the corresponding imidazolium salt monomer and solvent. As shown in Fig. S16 and Table S7,† varying the amount of H_2_O also results in a series BF_4_^–^ based MPILs.

A possible pore formation mechanism is proposed for our ionothermal polymerization. Rather than a homogeneous solvent, the slightly water-containing IL is regarded as having polar and nonpolar domains derived from imidazolium cations and alkyl chain aggregations, forming a nano-structural organization (Scheme 1B).^37^,^38^ The imidazolium salt monomer [C_1_DVIM]Br and initiator AIBN exist in the polar domain where polymerization occurs. The nonpolar domain functions as the template for the pore formation; after the reaction and removal of the IL solvent, the mesoporous polymer is formed. When the length of the alkyl chain is increased, the nonpolar domain becomes larger, causing expansion of the pore volume, which is well reflected in our data (Table S4†). The small amount of H_2_O tends to form larger hydrogen bonding-linked clusters and networks through H_2_O–anion interactions, enhancing the interaction between the imidazolium salt monomer [C_1_DVIM]Br and the IL solvent [C_4_MIM]Br.^39^

It is known that PILs feature an IL species in the monomeric repeating unit and incorporate the unique properties of ILs in their polymeric framework, giving rise to a new class of tunable polymeric materials that expand upon the properties and applications of ILs and common polyelectrolytes.^24^,^40^ Recently, studies of PILs have entered a rapidly extended growth phase, exhibiting more novel functions and applications.^8^,^25^,^41^,^42^ The building blocks and pore structures are all important for PILs especially in heterogeneous catalysis, inspiring the development of various pore formation strategies using different ionic frameworks. Nonetheless, up to now, only very limited success has been achieved on the pore formation of PILs derived from the homo-polymerization of an IL monomer alone (*i.e.*, without copolymerization with another non-IL monomer in order to create mesopores). MPILs with zwitterionic structures can be synthesized through a self-assembly process.^23^,^25^ Polycationic MPILs have the advantage of tunable anions and anion-exchanging properties, but only a handful of successful syntheses have been reported, usually relying on a soft or hard template.^9^,^10^,^26^ The systematic investigations described above prove that we have developed a new type of polycationic MPIL in this work. Both the specifically designed rigidly structured bis-vinylimidazolium salt monomer and the employed ionothermal route play crucial roles in the pore formation. The synthesis shows three major advantages: (1) avoiding the use of an external template and a volatile organic solvent; (2) enabling safe and simple operation at ambient pressure; (3) recycling of the IL solvent. The results give the first example of MPILs possessing extremely high densities of halogen anions (two per unit) coupled with high surface areas (up to 260 m^2^ g^–1^) and large pore volumes (up to 0.92 cm^3^ g^–1^).

**Fig. 3:**
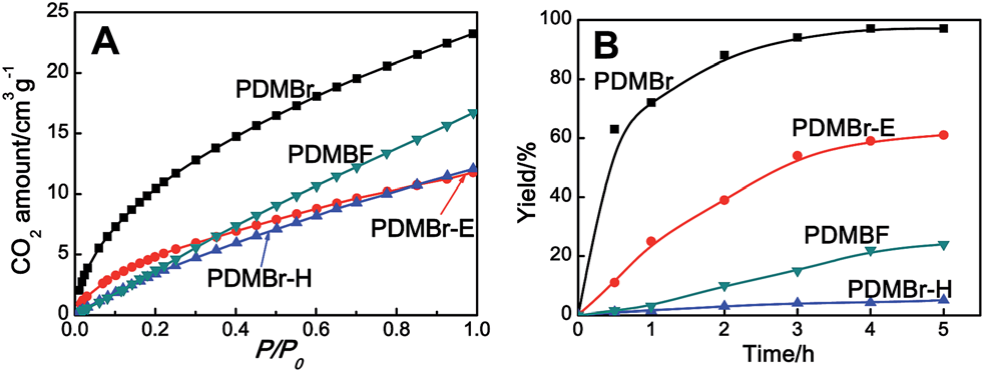
(A) CO_2_ adsorption isotherms at 273 K and (B) yield as a function of reaction time for the cycloaddition of CO_2_; reaction conditions: styrene oxide 10 mmol, catalyst 0.05 g (1.3 mol%), CO_2_ 1.0 MPa, 110 °C.

The obtained MPIL materials were engaged in CO_2_ adsorption and conversion to illustrate their specialty for practical utilizations. Halogen anions in ILs have been revealed to be active towards CO_2_ conversion. It is thus rational to expect that a nanoporous polymer with a large surface area and a halogen anion-enriched skeleton should be highly efficient for CO_2_ capture and conversion. The adsorption and catalytic conversion of CO_2_ were performed on the PDMBr, PDMBr-E, PDMBr-H and PDMBF samples. For CO_2_ adsorption, PDMBr shows a relatively high CO_2_ uptake of 1.02 mmol g^–1^ at 273 K and 1 atm (Fig. 3A), superior to those reported for other PIL materials under identical conditions^22^,^26^ and about two times the values for the nonporous counterparts (0.64 and 0.51 mmol g^–1^ for PDMBr-E and PDMBr-H). The result reveals the significant role of the large surface area of PDMBr for enhancing CO_2_ capture. In addition, an inferior CO_2_ uptake is observed for PDMBF than that for PDMBr, though they possess similar large surface areas, suggesting the positive effect of the Br^–^ anions in PDMBr for CO_2_ adsorption.

The cycloaddition of CO_2_ is one of the most promising strategies for the effective chemical fixation of CO_2_ (ref. 43–45) and the obtained product cyclic carbonates have important applications as aprotic organic solvents, electrolytes in Li-ion batteries, valuable precursors for polymers such as polycarbonates and polyurethanes, as well as raw materials for a wide range of reactions such as for the preparation of cosmetics and pharmaceuticals.^46^,^47^ The catalytic behaviours of the above four selected samples were assessed for the cycloaddition of CO_2_ into cyclic carbonates in the absence of any solvent, co-catalyst or other additives. The test starts from the conversion of styrene oxide and CO_2_ into styrene carbonate (Table 1, Fig. 3B and S17†). In mild conditions, the meso-macroporous PDMBr shows a rapid conversion rate and exhibits high conversion (99.0%) and selectivity (98.5%), comparable to the homogeneous imidazolium salt [C_1_DVIM]Br. A detailed comparison (Table S8†) reveals that PDMBr is among the most efficient IL-related heterogeneous catalysts for this reaction. In contrast, the bulky block PDMBr-H is inert in the reaction and the nonporous PDMBr-E, composed of small particles, also presents a much inferior conversion rate and activity (Fig. 3B). Considering the similar framework composition of the three samples, the high activity of PDMBr can be assigned to the abundant porosity, which promotes not only mass transfer but also the dispersion and accessibility of active sites. However, the porous analogue PDMBF exhibits an inferior conversion rate and activity (Fig. 3B), which indicates the beneficial role of the Br^–^ anions for the high activity of PDMBr. A six run recycling test shows that PDMBr can be steadily reused without a significant loss of activity (Fig. S18†), attributable to the well preserved structure in the reused catalyst (Fig. S19†). By evaluating the various epoxides (Table 2), PDMBr exhibits high yields for all of the target cyclic carbonates, indicative of good substrate compatibility. Notably, it is even very active for those extremely inert substrates, the aliphatic long carbon-chain alkyl epoxides, that are hardly converted by previous catalysts.^36^,^48^

**Table 2:** Cycloaddition of CO_2_ with various epoxides catalyzed by PDMBr^*a*^

Entry	Substrate	Product	*P*_CO_2__ (MPa)	Temp. (°C)	Time (h)	Con. (%)	Sel. (%)
1	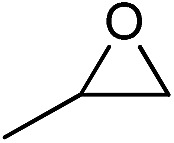	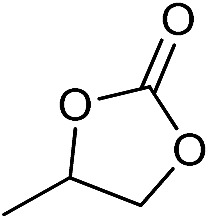	1	110	4	98.7	>99.9
2	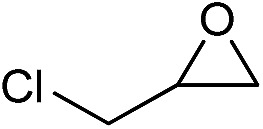	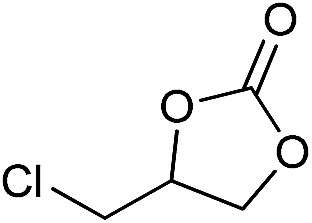	1	110	2	99.4	98.8^*b*^
3			0.1	120	12^*c*^	94.6	96.5^*b*^
4			0.1	70	48^*c*^	99.4	98.0^*b*^
5	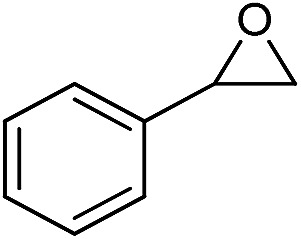	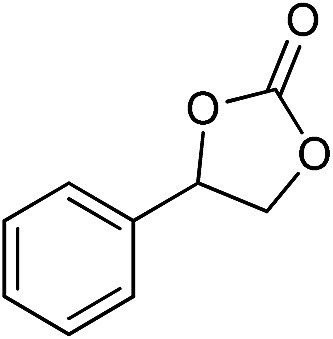	1	110	4	99.0	98.5^*d*^
6			0.1	120	12^*c*^	91.1	88.0^*d*^
7	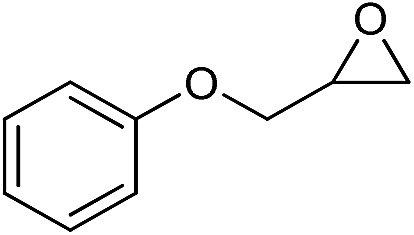	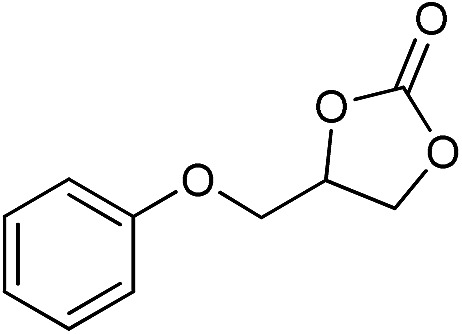	1	110	3	99.2	>99.9
8			0.1	120	12^*c*^	96.3	>99.9
9			0.1	70	48^*c*^	90.0	>99.9
10	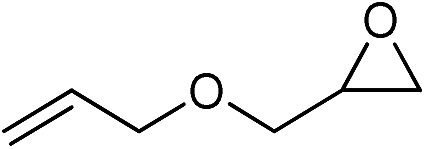	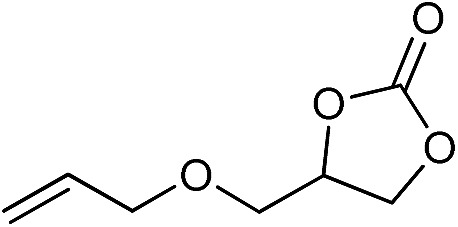	1	110	3	99.1	>99.9
11			1	110	1	93.7	>99.9
12			0.1	120	12^*c*^	98.7	>99.9
13			0.1	70	48^*c*^	95.4	>99.9
14	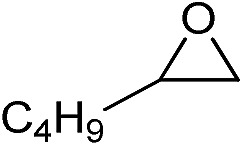	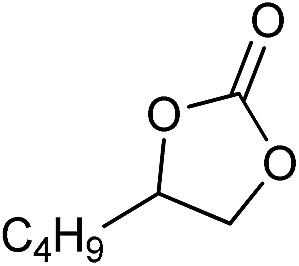	1	110	4	96.9	>99.9
15			0.1	90	48^*c*^	89.4	>99.9
16	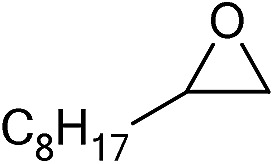	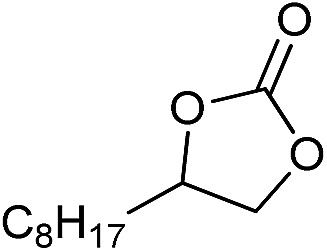	1	110	8	95.1	>99.9
17			0.1	120	48^*c*^	99.1	>99.9
18	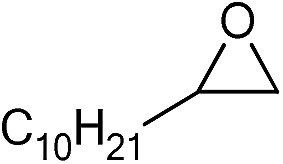	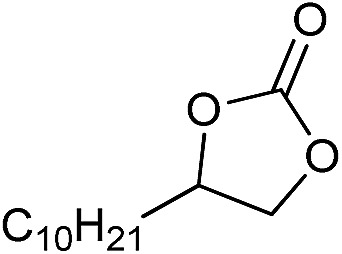	1	110	12	97.8	>99.9
19			0.1	120	48^*c*^	98.5	>99.9
20	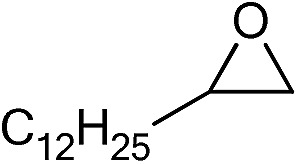	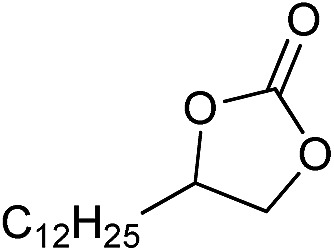	1	110	12	98.1	>99.9
21			0.1	120	48^*c*^	97.1	>99.9

^*a*^Reaction conditions: epoxide 10 mmol, catalyst 0.05 g (1.3 mol%).

^*b*^3-Chloro-1,2-propanediol was identified as the only by-product.

^*c*^Catalyst 0.1 g.

^*d*^Phenylacetaldehyde was identified as the only by-product.

Unprecedentedly, PDMBr can efficiently convert CO_2_ at atmospheric pressure (Table 2, entries 3, 6, 8, 12, 15, 17, 19 and 21), and even at the milder conditions of low temperature (70 °C, 0.1 MPa; entries 4, 9 and 13). Achieving good conversion of CO_2_ at mild conditions is still extremely difficult so far for a heterogeneous catalyst, though some homogeneous ILs^49^ and metal complexes^50^,^51^ exhibited excellent activity in the cycloaddition of CO_2_ to epoxides under much milder conditions, even at room temperature and atmospheric CO_2_ pressure.^52^,^53^ Actually, no report has appeared for a heterogeneous catalyst to promote the cycloaddition of CO_2_ to epoxides under ambient conditions without solvent or the addition of an external homogeneous co-catalyst. Metal–organic frameworks (MOFs) or metal-coordinated conjugated microporous polymers (CMPs) are active under ambient conditions only with external homogeneous co-catalysts (Table S9†). The metal-free polymeric material PP-Br required a higher temperature, such as 140 °C, to efficiently convert glycidyl phenyl ether when DMF was used as the solvent.^54^ In contrast, our synthesized PDMBr catalyst exhibits the high yield of 90% for the same substrate at the lower temperature of 70 °C under solvent-free conditions. Various other substrates, including the inert long carbon-chain alkyl epoxides, can be converted by PDMBr into the corresponding cyclic carbonates with high yields under ambient conditions. Some epoxides, such as glycidyl phenyl ether, allyl glycidyl ether and epichlorohydrin, can be efficiently converted even at the mild conditions of atmospheric pressure and the low temperature of 70 °C (entries 4, 9 and 13). In short, previous results relative to the heterogeneous cycloaddition of CO_2_ under mild conditions, though rarely reported, still relied on adding either a solvent or a co-catalyst (Table S9†). Our newly synthesized MPIL is the only metal–solvent–additive free heterogeneous catalyst for cycloaddition of CO_2_ that performs well under such mild conditions. The high activity can be attributed to: (1) the large surface area and specific hierarchical meso-macroporous structure that enables the good dispersion of active sites (Br^–^) and accelerates the mass transfer of the larger molecules of substrates and products; (2) the good intrinsic CO_2_-philicity (maybe arising from the enriched Br^–^ ions in the large-surface-area pore structure) improves the local concentration of CO_2_ around the catalytic centers inside the pores of the polymeric framework.

## Conclusions

In summary, we report a facile, atom-efficient, green and sustainable ionothermal synthesis towards tunable MPILs materials with high specific surface areas. Meso-macroporous hierarchical polymers are achieved from this template-free radical self-polymerization process. The newly designed structurally rigid bis-vinylimidazolium salt monomer dominates this synthesis. Owing to the abundant porosity with enriched Br^–^ anions, the obtained MPIL shows enhanced CO_2_ adsorption and conversion capacities. It is the first example of a metal–solvent–additive free recyclable catalyst for the highly efficient heterogeneous cycloaddition of CO_2_ at atmospheric pressure and low temperatures, and is even very active for the extremely inert long carbon-chain epoxides. This work provides a novel heterogeneous candidate for CO_2_ conversion, which may trigger an attractive outline for CO_2_ conversion at mild conditions down to atmospheric pressure and even lower temperatures.

## Experimental

### Chemicals and materials

All reagents were of analytical grade and used as purchased without further purification. [C_*n*_MIM]Br (*n* = 2, 4, 6; ≥99% purity), [C_4_MIM]BF_4_ (≥99% purity), and [P_4444_]Br (≥99% purity) were provided by Lanzhou Greenchem ILS, LICP, Chinese Academy of Sciences. CO_2_ (>99.95% purity) was provided by the Gas Supply Center of Nanjing SanLe Group Co., Ltd.

### Characterization

^1^H NMR spectra and ^13^C NMR spectra were collected on a Bruker DPX 500 spectrometer at ambient temperature using DMSO or D_2_O as the solvent and TMS (tetramethylsilane) as the internal reference. Scanning electron microscopy (SEM) images were obtained on a Hitachi S-4800 field-emission scanning electron microscope (accelerated voltage: 5 kV). Transmission electron microscopy (TEM) analysis was performed on a JEM-2100 (JEOL) electron microscope operating at 200 kV. The nitrogen sorption isotherms and pore size distribution curves were measured at the temperature of liquid nitrogen (77 K) using a BELSORP-MINI analyzer. The samples were degassed at 120 °C for 3 h before analysis. The mesopore size of the PILs was calculated from the adsorption branch by using the Barrett–Joyner–Halenda (BJH) model. The macropore size distribution was detected using a Poremaster GT-60 mercury inclusion porosimeter. Fourier transform infrared spectroscopy (FTIR) spectra were recorded on an Agilent Carry 660 FT-IR instrument (KBr disks) ranging from 4000 to 400 cm^–1^. X-ray photoelectron spectroscopy (XPS) was conducted on a PHI 5000 Versa Probe X-ray photoelectron spectrometer equipped with Al Kα radiation (1486.6 eV). The C 1s peak at 285.2 eV was used as the reference for the binding energies. Elemental analyses were performed on a CHN elemental analyzer Vario EL cube. Thermogravimetric (TG) analysis was performed with an STA409 instrument in a dry air atmosphere at a heating rate of 10 °C min^–1^. Solid state ^13^C NMR spectra were recorded on a Bruker AVANCE-III spectrometer in a magnetic field strength of 9.4 T, corresponding to the Larmor frequency of 400 MHz for ^13^C nuclei, with a CP/MAS unit at room temperature. The spinning rate and the contact time were 12 KHz and 2.5 ms, respectively. The pulse width, spectral width, and acquisition time were 2.50 μs, 300.0 KHz, and 33.91 ms. 10 000 scans were accumulated for each spectrum. XRD patterns were collected on a Smart Lab diffractometer from Rigaku equipped with a 9 kW rotating anode Cu source at 45 kV and 200 mA from 5° to 50° with a scan rate of 0.2° s^–1^. CO_2_ adsorption isotherms were measured at 273 K on a Micromeritics ASAP 2020 volumetric adsorption analyzer. Prior to the measurements, the samples were degassed at 100 °C for 12 h.

### Synthesis of the bis-vinylimidazolium and mono-vinylimidazolium salts

The bis-vinylimidazolium salts [C_1_DVIM]Br, [C_2_DVIM]Br and [C_4_DVIM]Br (Scheme 2A) were conveniently obtained *via* the reaction between 1-vinylimidazole and dibromomethane (CH_2_Br_2_), 1,2-dibromoethane ((CH_2_)_2_Br_2_) and 1,4-dibromobutane ((CH_2_)_4_Br_2_), respectively, in a Teflon-lined stainless steel autoclave. Typically, 1-vinylimidazole (5.00 g, 53.2 mmol) and CH_2_Br_2_ (4.62 g, 26.6 mmol) were dissolved in 5 mL THF. After stirring at room temperature for 1 h, the mixture was solvothermally treated at 100 °C for 24 h. After cooling to room temperature, the obtained crude salt was washed with diethyl ether (5 × 100 mL) and dried under vacuum, giving the light yellow powder product, 3,3-methylene-divinylimidazole dibromide.

#### [C_1_DVIM]Br

Yield: 7.8 g, 81%. ^1^H NMR (300 MHz, DMSO, TMS) *δ* (ppm) = 10.06 (s, 1H), 8.39 (d, 2H), 7.47 (q, 1H), 6.89 (s, 1H), 6.08 (q, 1H), 5.51 (q, 1H). ^13^C NMR (75.5 MHz, DMSO) *δ* (ppm) = 137.1, 128.8, 122.8, 119.5, 110.0, 58.1.

#### [C_2_DVIM]Br

Yield: 89%. ^1^H NMR (300 MHz, DMSO, TMS) *δ* (ppm) = 9.82 (s, 1H), 8.28 (s, 1H), 7.98 (s, 1H), 7.39 (q, 1H), 6.03 (d, 1H), 5.44 (d, 1H), 4.93 (s, 2H). ^13^C NMR (75.5 MHz, DMSO) *δ* (ppm) = 136.1, 128.8, 123.3, 119.5, 109.3, 48.4.

#### [C_4_DVIM]Br

Yield: 91%. ^1^H NMR (300 MHz, DMSO, TMS) *δ* (ppm) = 9.92 (s, 1H), 8.36 (s, 1H), 8.09 (s, 1H), 7.47 (m, 1H), 6.07 (d, 1H), 5.40 (d, 1H), 4.35 (s, 2H), 1.89 (s, 2H). ^13^C NMR (75.5 MHz, DMSO) *δ* (ppm) = 135.3, 128.7, 123.2, 119.3, 108.3, 48.3, 26.2.

#### [C_1_DVIM]BF_4_

[C_1_DVIM]BF_4_ was prepared from the anion exchange of [C_1_DVIM]Br with BF_4_^–^. [C_1_DVIM]Br (1.81 g, 5 mmol) was dissolved in water (50 mL), followed by the addition of AgBF_4_ (1.95 g, 10 mmol), resulting in the appearance of a precipitated salt. The mixture was stirred at room temperature for 1 h. After filtration, the filtrate was evaporated to give the product as a gray solid. The product [C_1_DVIM]BF_4_ was dried in a vacuum at 80 °C for 12 h. Yield: 1.6 g, 85%. ^1^H NMR (300 MHz, DMSO, TMS) *δ* (ppm) = 9.61 (s, 1H), 8.27 (d, 2H), 7.44 (q, 1H), 6.67 (s, 1H), 6.02 (d, 1H), 5.54 (d, 1H). ^13^C NMR (75.5 MHz, DMSO) *δ* (ppm) = 137.2, 128.8, 122.9, 120.0, 110.0, 58.8.

#### [C_1_VIM]Br

[C_1_VIM]Br was synthesized consulting the previous literature.^55^ 1-Vinylimidazole (5.00 g, 53.2 mmol) and CH_3_CH_2_Br (5.80 g, 53.2 mmol) were placed in a 50 mL round-bottom flask and the mixture was refluxed under vigorous stirring at 60 °C for 24 h. Then, the obtained white solid was filtered, followed by washing with diethyl ether several times and vacuum drying at 80 °C for 24 h to give the final product. Yield: 92%. ^1^H NMR (300 MHz, DMSO, TMS) *δ* (ppm) = 9.93 (s, 1H), 8.36 (s, 1H), 8.09 (s, 1H), 7.42 (q, 1H), 6.09 (d, 1H), 5.39 (d, 1H), 4.31 (q, 2H), 1.44 (t, 3H). ^13^C NMR (75.5 MHz, DMSO) *δ* (ppm) = 135.0, 128.7, 122.9, 119.2, 108.7, 44.6, 14.9.

### Synthesis of the PIL materials

The mesoporous poly(ionic liquid)s (MPILs) were synthesized through the free radical self-polymerization of bis-vinylimidazolium or mono-vinylimidazolium salts, in which an ionic liquid was used as the solvent and a small amount water was added if necessary. Typically (Scheme 1A), [C_1_DVIM]Br (0.3 g), [C_4_MIM]Br (6 g), H_2_O (0.75 mL) and AIBN (0.03 g, 10 wt%, according to the amount of [C_1_DVIM]Br) were placed in a reaction tube. Subsequently, the above mixture was kept stirring for 2 h until a homogeneous and transparent solution was achieved. The polymerization was triggered by statically heating at 100 °C in an oil bath at atmospheric pressure for 24 h. The solidified composite was washed with deionized water and then dried under vacuum at 80 °C. The yield of the MPIL product was around 97% and the solvent IL [C_4_MIM]Br was recovered quantitatively (>99% yield) by evaporation of the water to give a purity as high as the fresh one, as indicated by the ^1^H NMR spectrum (Fig. S7†). The other PIL or MPIL materials were prepared using similar precursors by varying the monomers ([C_1_DVIM]BF_4_, [C_2_DVIM]Br, [C_4_DVIM]Br or [C_2_VIM]Br), solvents (H_2_O, EtOH, AcOH, DMF or DMSO) or the imidazolium salts ([C_*n*_MIM]Br (*n* = 2, 6, 8), [C_4_Py]Br or [P_4444_]Br) (Scheme 2B).

The detailed syntheses of PDMBr-E and PDMBr-H are as follows.

#### PDMBr-E

The bis-vinylimidazolium salt monomer [C_1_DVIM]Br (0.3 g) and 0.03 g of the initiator AIBN were dissolved in the solvent mix of EtOH/H_2_O (7 g, 6 : 1 w/w) and the mixture was stirred to form a homogeneous solution. The polymerization process was not stirred and was triggered in the presence of the initiator by heating at 100 °C in an oil bath under atmosphere for 24 h. The obtained product was washed with deionized water several times and dried under vacuum at 80 °C. The yield of the product PDMBr-E was around 75%.

#### PDMBr-H

The bis-vinylimidazolium salt monomer [C_1_DVIM]Br (0.3 g) and AIBN (0.03 g) were dissolved in H_2_O (0.75 g). The obtained product was washed with deionized water several times and dried under vacuum at 80 °C. The yield of the product PDMBr-H was around 65%.

### Typical procedure for the synthesis of a cyclic carbonate from an epoxide and CO_2_

The variable pressure catalytic conversion of CO_2_ to cyclic carbonate was carried out using a stainless-steel autoclave, while the ambient CO_2_ conversion was operated in a 25 mL glass flask refluxing with a CO_2_ balloon. In a typical catalytic cycloaddition, styrene oxide (SO, 10 mmol) and the catalyst PDMBr (0.05 g, 1.3 mol%, according to the amount of ionic liquid) were placed in a 25 mL stainless-steel autoclave equipped with a magnetic stirrer. After being sealed, the autoclave was carefully flushed once with CO_2_. The reaction was carried out at the specified temperature and CO_2_ pressure for the desired period of time. After the reaction, the reactor was cooled in an ice-water bath and slowly depressurized. The internal standard, *n*-dodecane (0.5 g), was added and the resulting mixture was diluted with ethyl acetate. The reaction mixture was centrifuged to remove the solid catalyst, and the liquid was analyzed using a gas chromatograph (Agilent 7890B) equipped with a FID detector and a capillary column (HP-5, 30 m × 0.25 mm × 0.25 μm). The carrier gas was N_2_ with a flow of 1 mL min^–1^. The injector temperature was 250 °C, while the column temperature was maintained for 2 min at 100 °C and then heated by 10 °C min^–1^ to 220 °C, where it was maintained for 2 min. GC-MS (Bruker Scion 436 GC-MS) equipped with a capillary column (BR-5 MS 15 m × 0.25 mm × 0.25 μm) was also used to confirm the composition of the cycloaddition products. The conversion over various substrates was tested under both the moderate pressure of 1 MPa and atmospheric pressure. The reusability was tested in six-run cycling experiments. The solid catalyst was separated by filtration, washed, dried, and then charged into the next run for reuse.

## Supplementary Material

Supplementary information
