# Supplementary material for: Heterogeneous conversion of CO2 into cyclic carbonates at ambient pressure catalyzed by ionothermal-derived meso-macroporous hierarchical poly(ionic liquid)s
Source: Chem Sci. 2015 Aug 27;6(12):6916–24. doi: 10.1039/c5sc02050f (PMC5951110; doi:10.1039/c5sc02050f)
Supplement: Supplementary file 1 [file SC-006-C5SC02050F-s001.pdf]

Electronic Supplementary Material (ESI) for Chemical Science.
This journal is © The Royal Society of Chemistry 2015

# Supporting Information

## Heterogeneous conversion of $CO_2$ into cyclic carbonates at ambient pressure catalyzed by ionothermal-derived meso-macroporous hierarchical poly(ionic liquid)s

Xiaochen Wang†, Yu Zhou†, Zengjing Guo, Guojian Chen, Jing Li, Yuming Shi, Yangqing Liu, Jun Wang*

*State Key Laboratory of Materials-Oriented Chemical Engineering, College of Chemistry and Chemical Engineering, Nanjing Tech University (Former Nanjing University of Technology), Nanjing 210009, China*

*Corresponding author. Tel: +86-25-83172264, Fax: +86-25-83172261, E-mail: junwang@njtech.edu.cn (J. Wang)*

† The authors contributed equally to this work.

### The details of IR and $^{13}C$ NMR spectra

As shown in the FT-IR spectra (Figure 2C), all the samples as well as the bis-vinylimidazolium salt [$C_1$DVIM]Br exhibit a very broad and strong band at 3000-2850 $cm^{-1}$ attributable to stretching vibrations of the C-H group.[1] The samples PDMBr-E, PDMBr-H and PDMBr all present the featured bands for imidazole ring at ca. 1647 and 1568 $cm^{-1}$, evidencing the existence of the imidazolium moiety in the polycationic framework.[2,3,] In addition, the bands around 962 $cm^{-1}$ attributed to the unsaturated C-H vibrations of vinyl groups are clearly observed on the spectrum of [$C_1$DVIM]Br but disappears on the spectra of PDMBr-E, PDMBr-H and PDMBr, suggesting the consuming of unreacted vinyl groups.[4] The results demonstrate the successful polymerization of the bis-vinylimidazolium salt monomer with high polymerization degree. Besides, PDMBr-E, PDMBr-H and PDMBr show similar spectra, indicating they have the same chemical composition of the polymeric skeleton. Importantly, PDMBr prepared in IL [$C_4$MIM]Br shows the similar spectra with PDMBr-E and PDMBr-H, confirming the successful removal of IL solvent [$C_4$MIM]Br from the polymer in the washing step.

Figure 2D depicts the solid state $^{13}C$ NMR spectra of PDMBr-E, PDMBr-H and PDMBr. Almost same peaks in $^{13}C$ NMR spectra are observed over these three samples. The chemical shift peak at approximately 42 ppm corresponds to terminal $CH_2$. The peak at around 55 ppm is attributed to CH moiety and the adjacent peak at around 60 ppm corresponds to the methylene units linking the two imidazolium rings. The strong overlapping peaks at around 125 ppm are ascribed to the carbon atoms of the C4 and C5 atoms in imidazolate ring. The C2 atom in the same imidazolate ring is reflected by the single one at 138 ppm.[5] The almost same NMR spectra over the samples PAMBr, PDMBr-E and PDMBr-H further illustrate the same chemical framework over these polymers, agreeing with the results of FT-IR spectra.

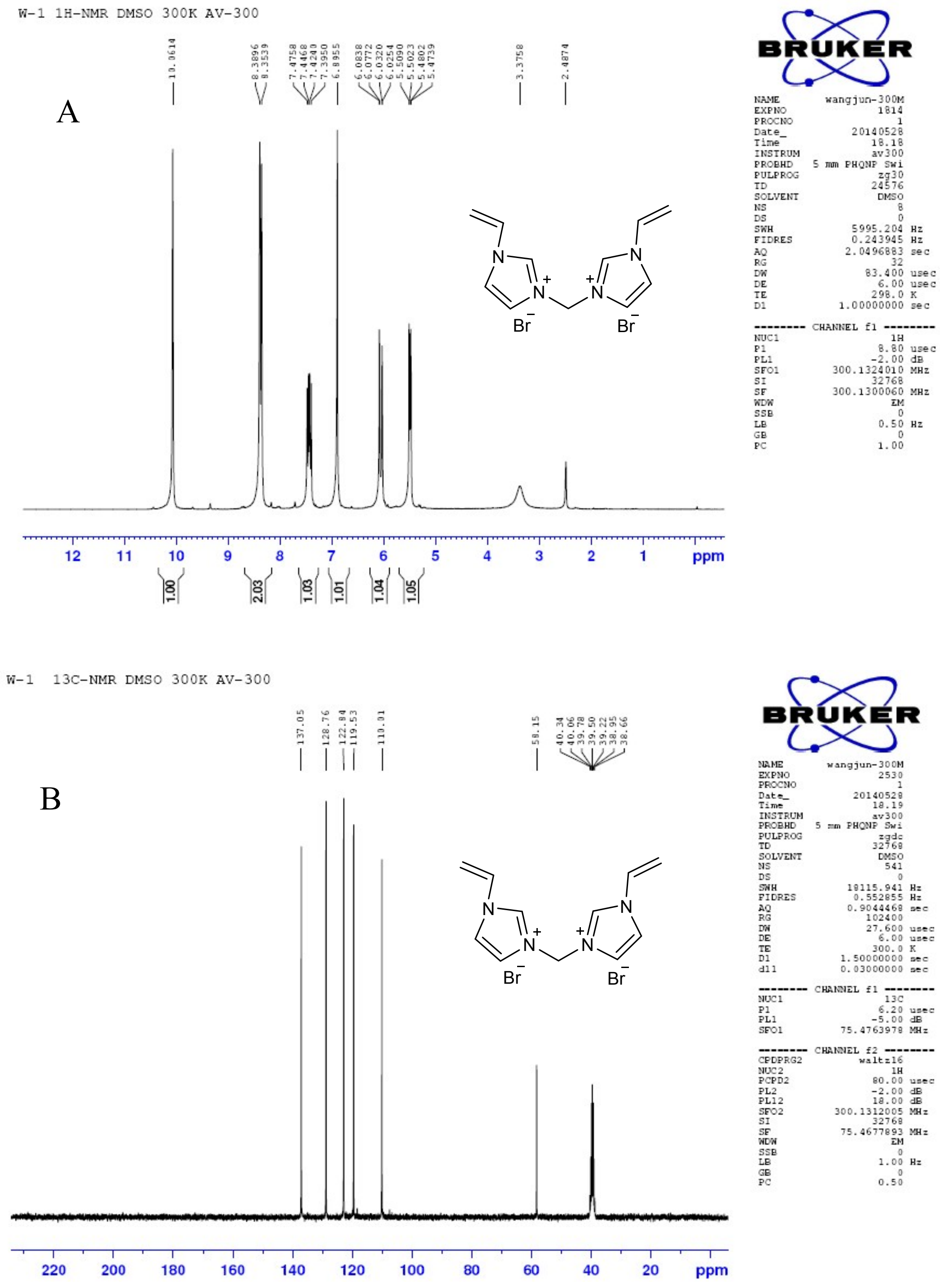


**Figure S1. (**A) $^{1}$H NMR and (B) $^{1}$C NMR of [$C_1$DVIM]Br.

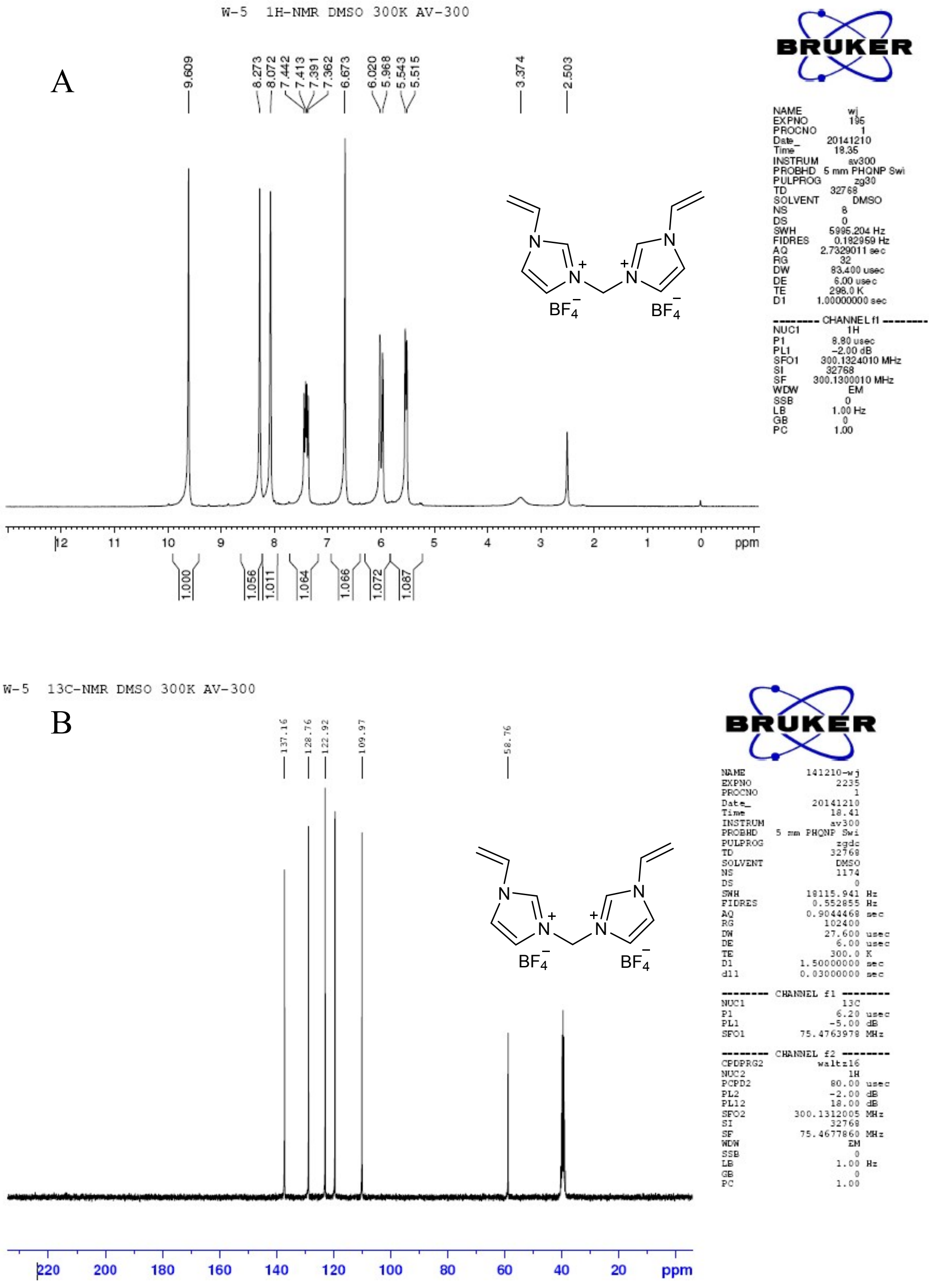


**Figure S2.** (A) $^{1}$H NMR and (B) $^{1}$C NMR of $[C_1DVIM]BF_4$.

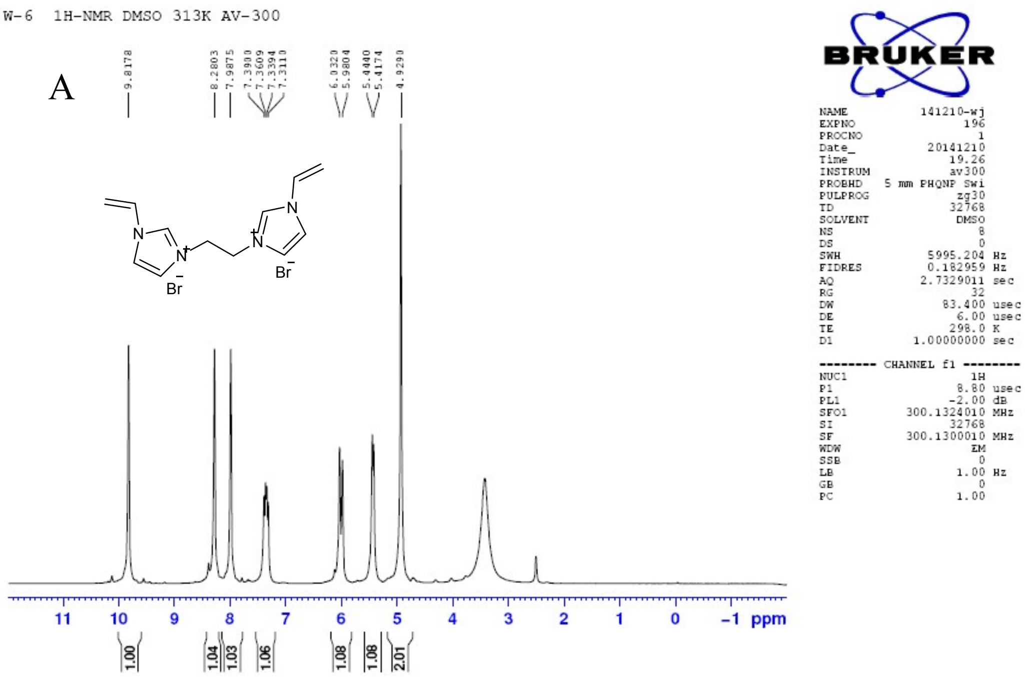


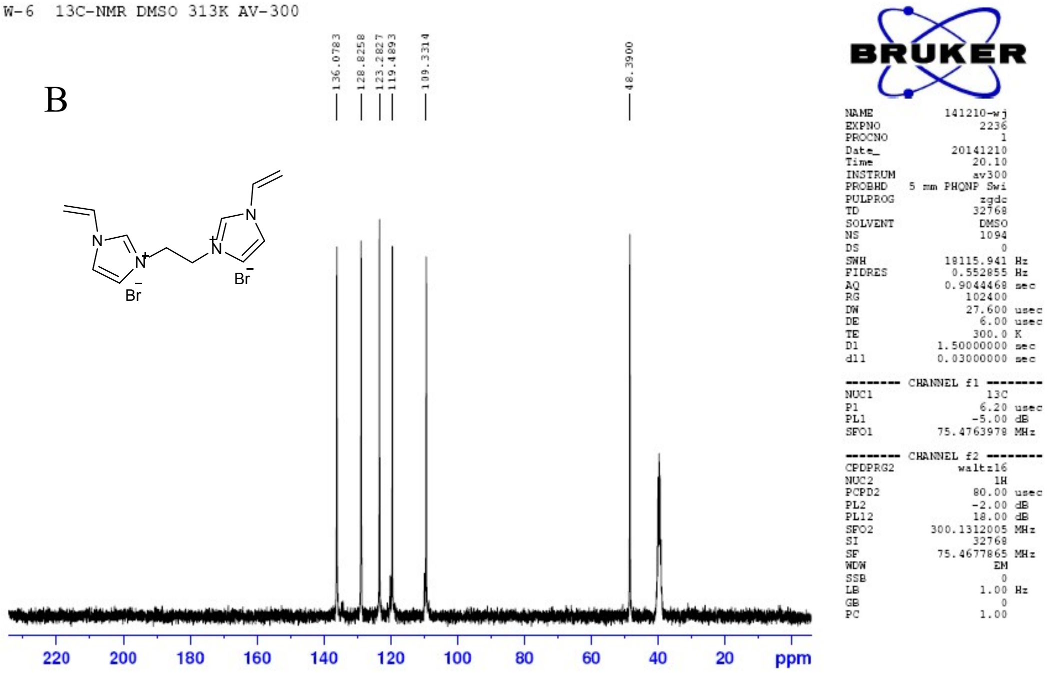


**Figure S3.** (A) $^{1}$H NMR and (B) $^{1}$C NMR of [$C_2$DVIM]Br.

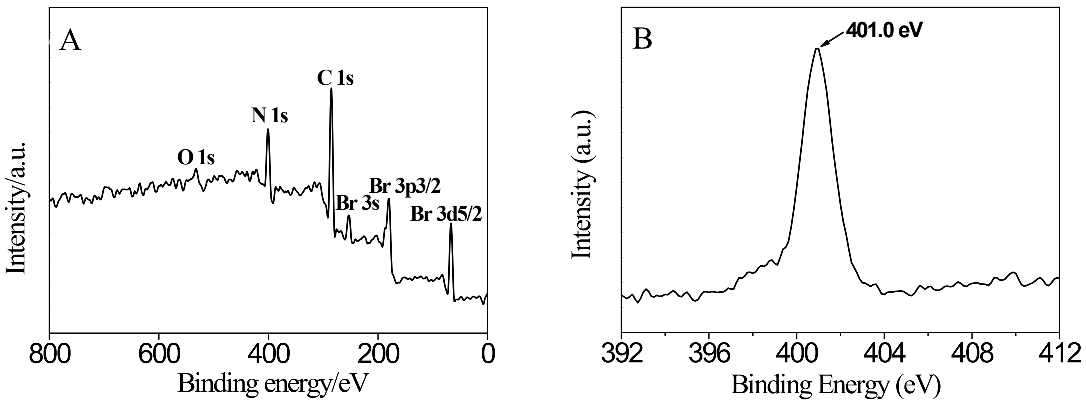


**Figure S4.** (A) Full XPS spectrum and (B) the high resolution of N 1s XPS spectrum for PAMBr.

The peaks corresponding to C 1s, N 1s and O 1s can be clearly observed in the XPS full spectrum, indicating that the polycationic chemical components is carbon and nitrogen, accompanied by a small amount of oxygen come from the adsorbed water (Figure S4A). The bromine element has also been detected in the XPS survey of PAMBr, revealing the existence of abudunt bromine anion site in the polymer structure.[6] The high-resolution N 1s XPS spectrum with the peak at 401.0 eV is assigned to C-N bonds in imidazolate ring (Figure S4B).[3]

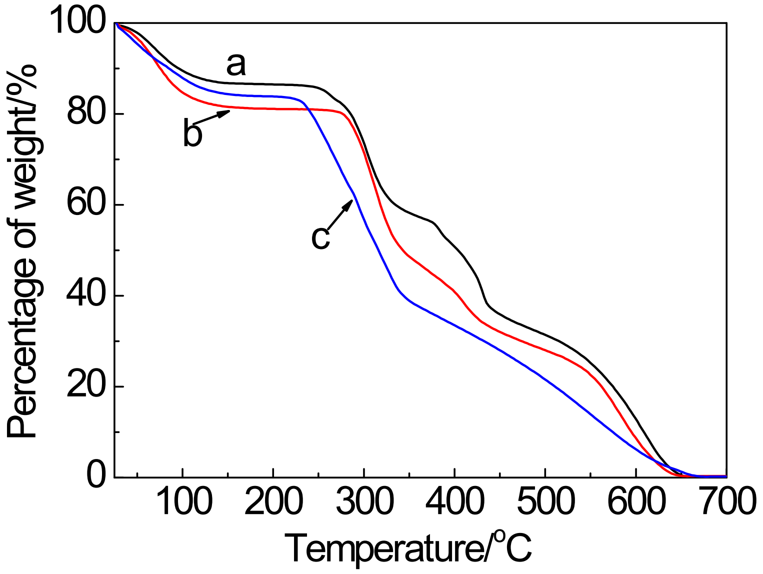


**Figure S5.** TG curves of (a) PDMBr, (b) PDMBr-H and (c) PDMBr-E.

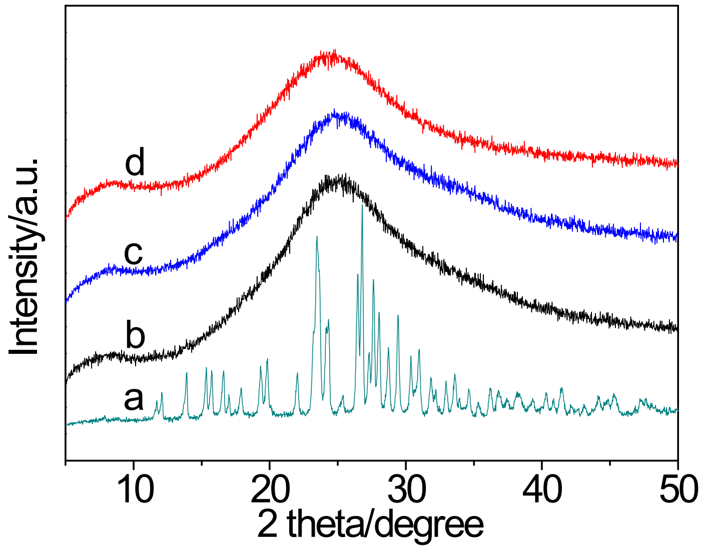


**Figure S6.** XRD patterns of (a) [$C_1$DVIM]Br, (b) PDMBr, (c) PDMBr-H and (d) PDMBr-E.

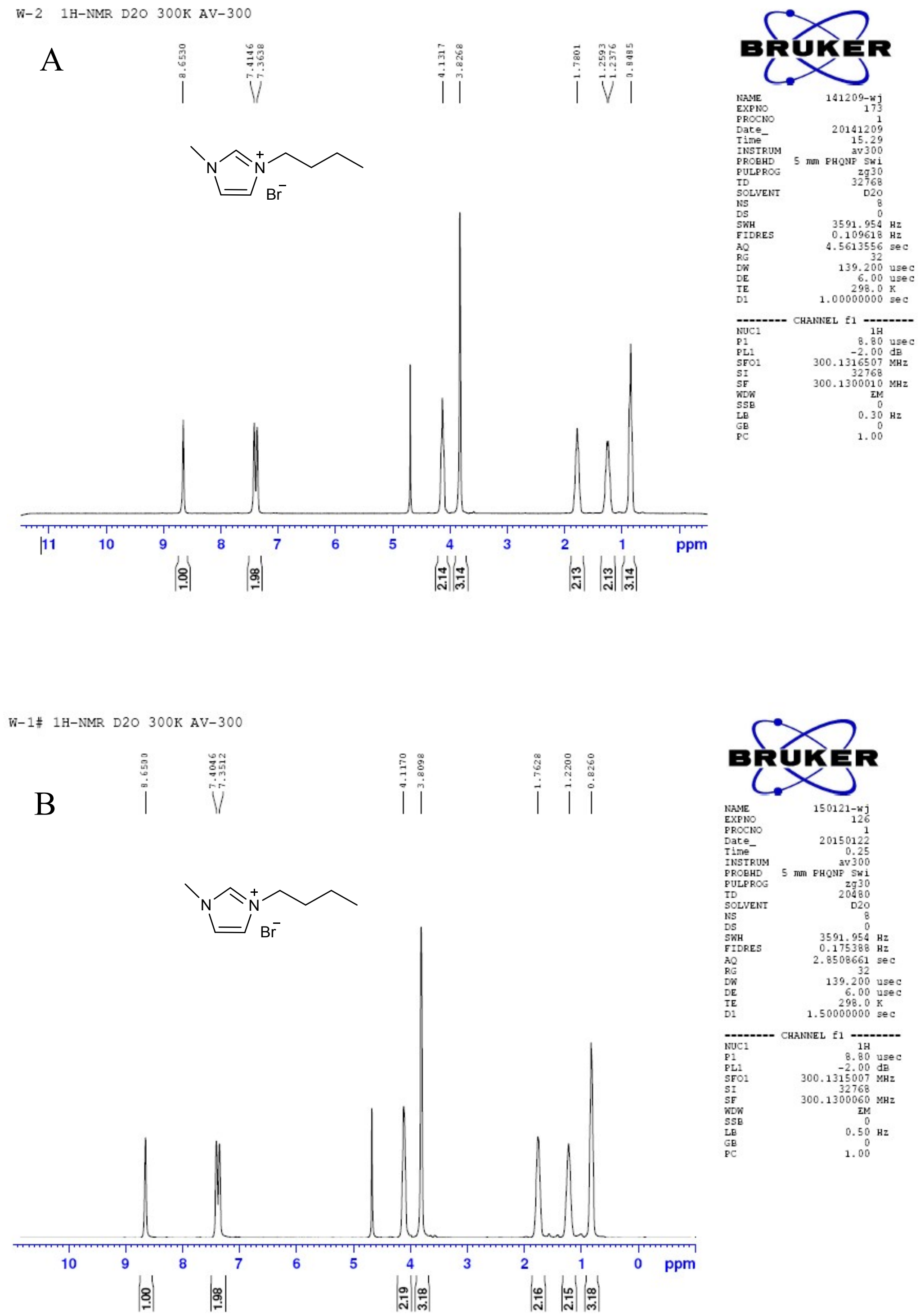


**Figure S7.** $^1$H NMR of (A) fresh [$C_4$MIM]Br and (B) recovered [$C_4$MIM]Br.

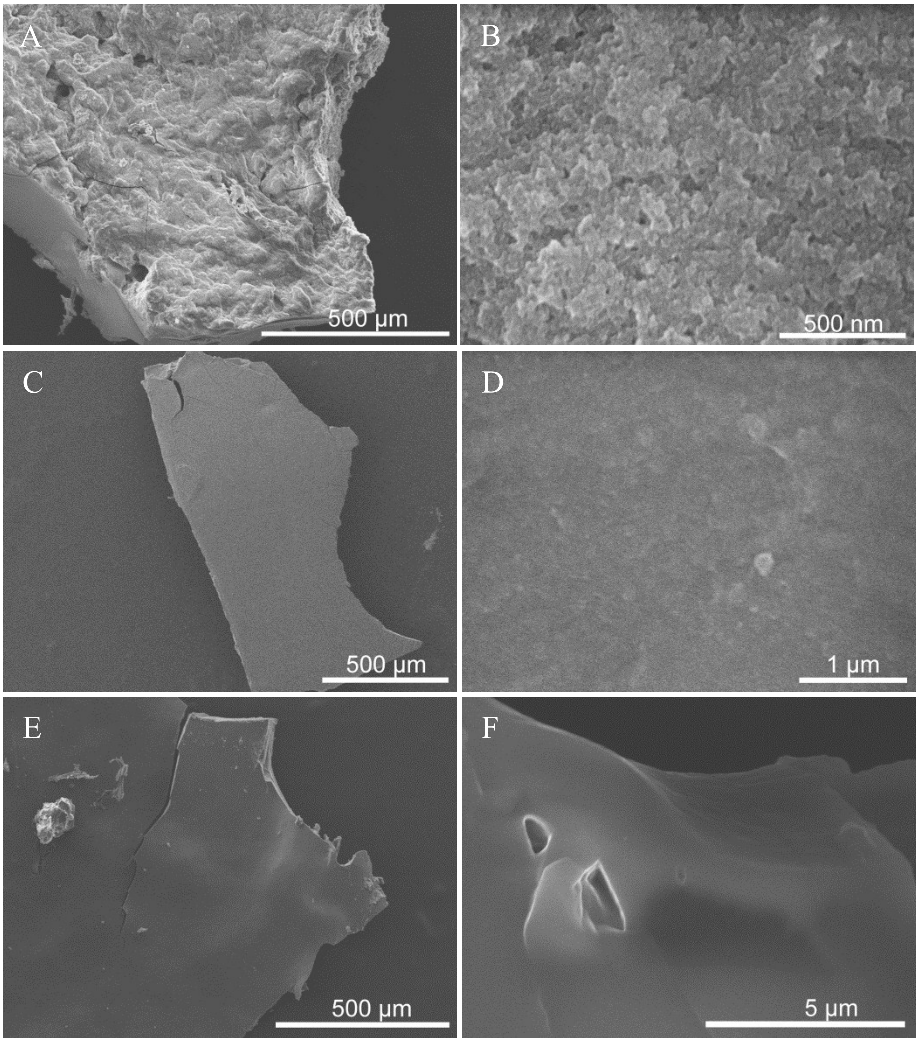


**Figure S8.** SEM images of the samples prepared with the initial mass composition of imidazolium salt monomer 0.3 g, [$C_4$MIM]Br 6 g, $H_2O$ 0.75 g and AIBN 0.03 g. Imidazolium salt monomer: (A, B) [$C_2$DVIM]Br; (C, D) [$C_4$DVIM]Br; (E, F) [$C_2$VIM]Br.

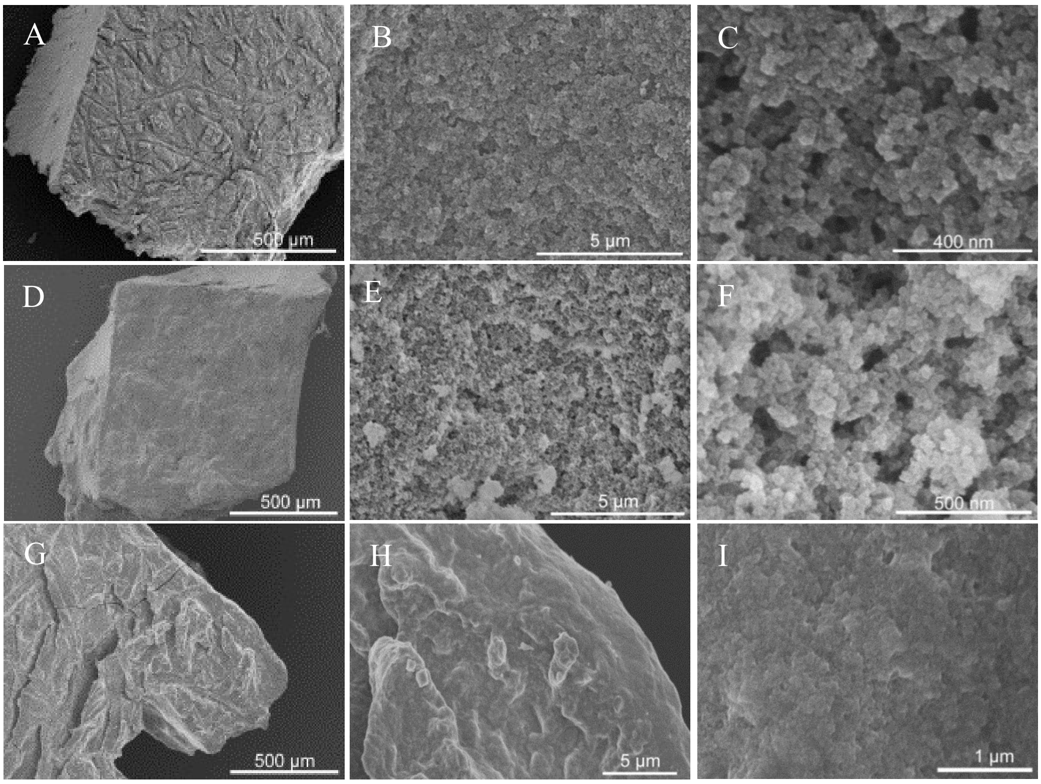


**Figure S9.** SEM images of the samples prepared with the initial mass composition of [$C_1$DVIM]Br 0.3 g, IL 6 g, $H_2O$ 0.75 g and AIBN 0.03 g. IL: (A, B, C) [$C_2$MIM]Br; (D, E, F) [$C_6$MIM]Br; (G, H, I) [$C_8$MIM]Br.

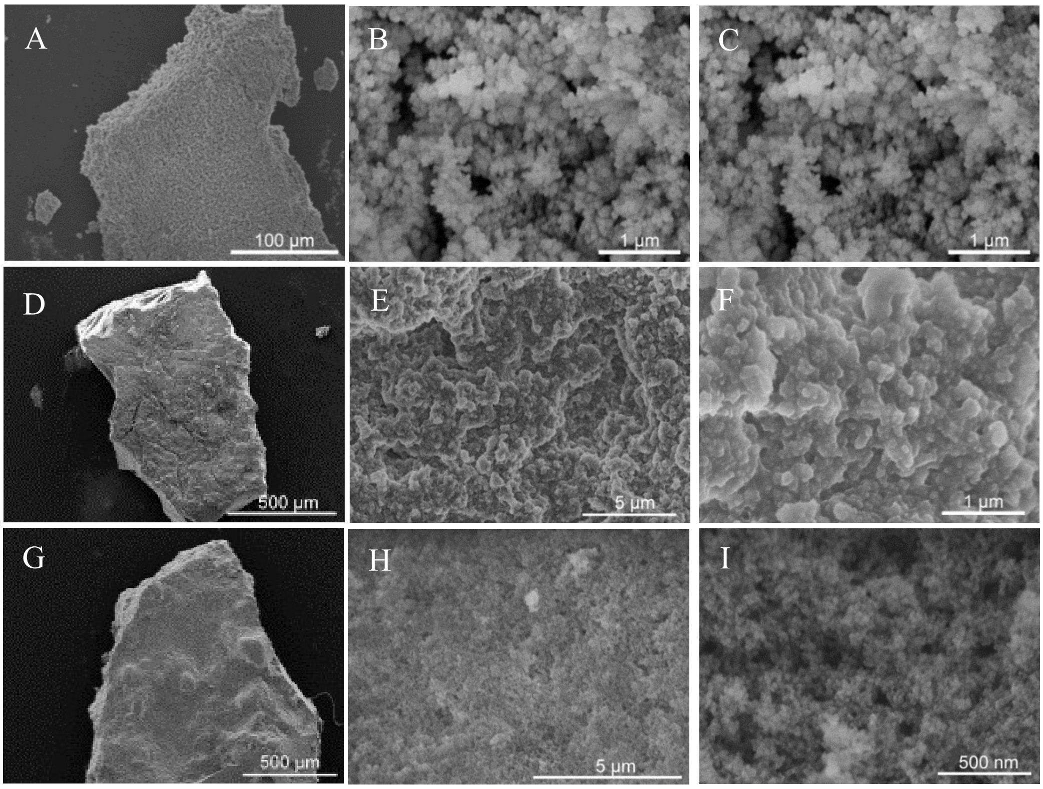


**Figure S10.** SEM images of the samples prepared with the initial mass composition of $[C_1DVIM]Br$ 0.3g, IL/ionic salt 6 g, $H_2O$ 0.75 g and AIBN 0.03 g. IL/ionic salt: (A, B, C) $[P_{4444}]Br$, (C, D, E) TPABr and (F, G, H) TBABr.

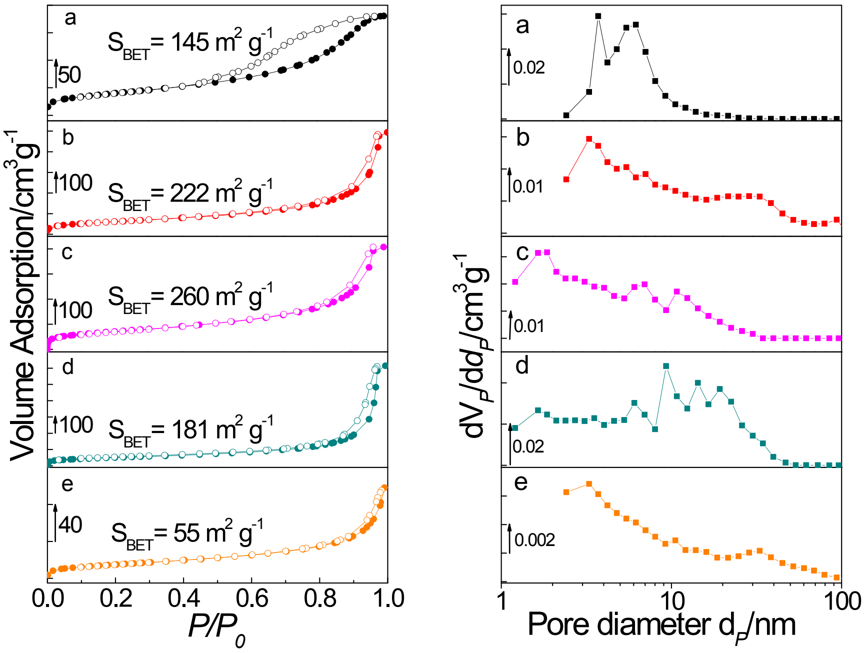


**Figure S11.** $N_2$ sorption isotherms (left) and the corresponding pore size distribution curves (right) of the samples prepared with the initial mass composition of [$C_1$DVIM]Br 0.3 g, IL 6 g and $H_2O$ 0.75 g. IL/ionic salt: (a) [$C_2$MIM]Br, (b) [$C_6$MIM]Br, (c) TPABr, (d) TBABr and (e) [$P_{4444}$]Br.

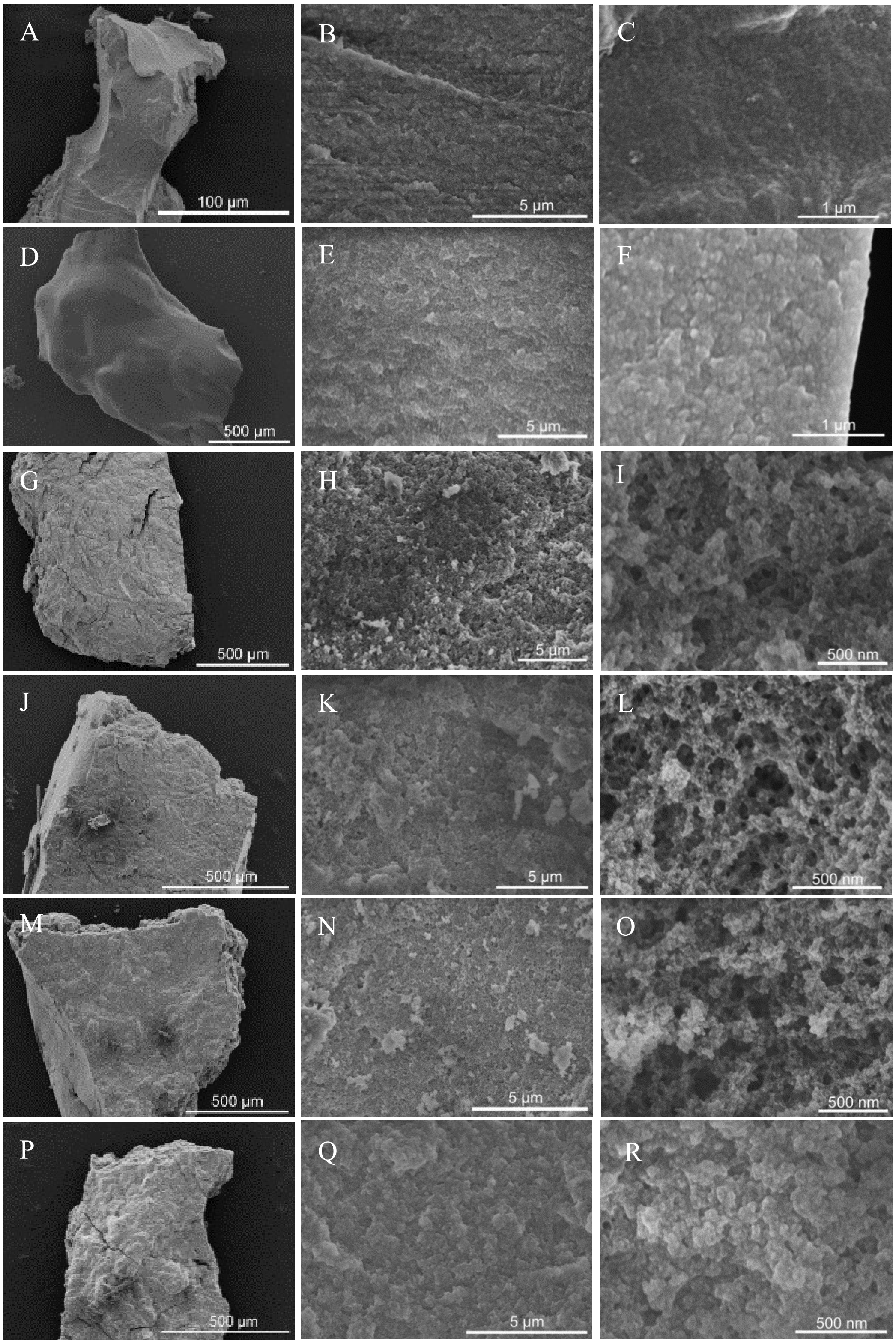


**Figure S12.** SEM images of the samples prepared with the initial mass composition of [$C_1$DVIM]Br 0.3 g, [$C_4$MIM]Br 6 g, $H_2O$ *x* g and AIBN 0.03 g. (A, B, C) *x*=0; (D, E, F) *x*=0.25; (G, H, I) *x*=0.5; (J, K, L) *x*=1; (M, N, O) *x*=1.5; (P, Q, R) *x*=2.

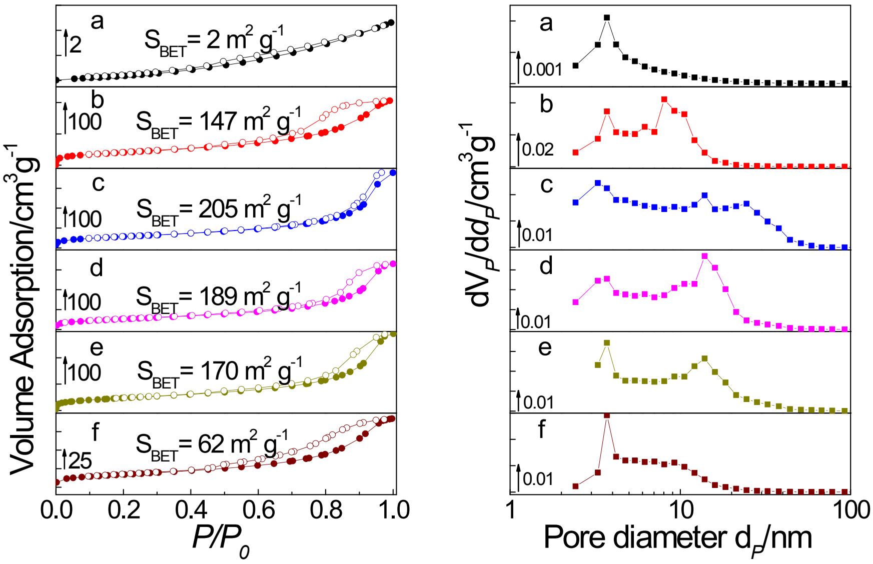


**Figure S13.** $N_2$ sorption isotherms (left) and the corresponding pore size distribution curves (right) of the samples prepared with the initial mass composition of [$C_1$DVIM]Br 0.3 g, [$C_4$MIM]Br 6 g, $H_2O$ $x$ g and AIBN 0.03 g. (a) $x$=0.25; (b) $x$= 0.5; (c) $x$= 0.75; (d) $x$= 1; (e) $x$= 1.5, and (f) $x$= 2.

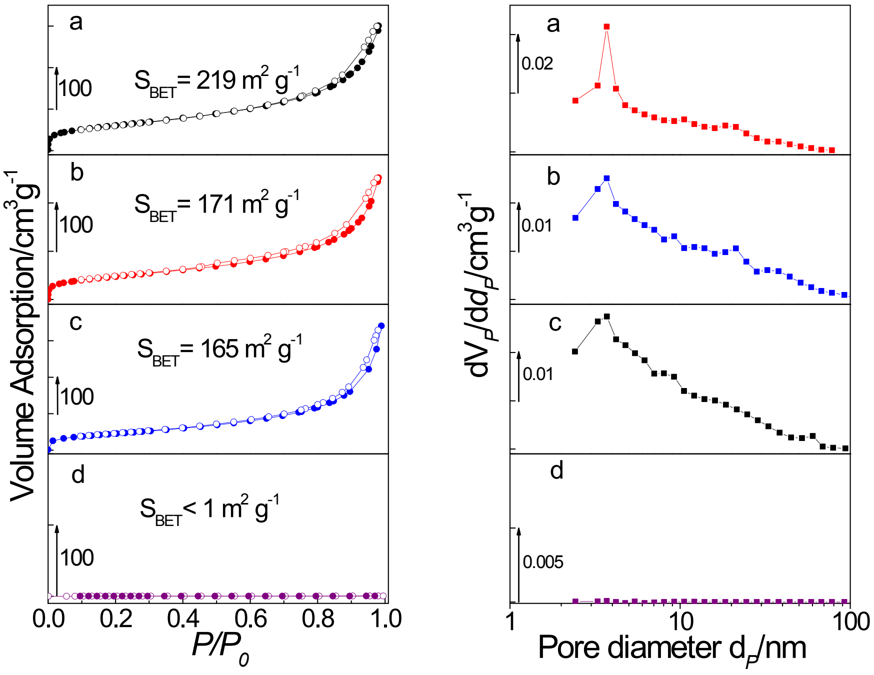


**Figure S14.** $N_2$ sorption isotherms (left) and the corresponding pore size distribution curves (right) of the samples prepared with the initial mass composition of [$C_1$DVIM]Br 0.3 g, [$C_4$MIM]Br $x$ g, $H_2O$ 0.75 g and AIBN 0.03 g. (a) $x$=3, (b) $x$=2, (c) $x$=1 and (d) $x$=0.5.

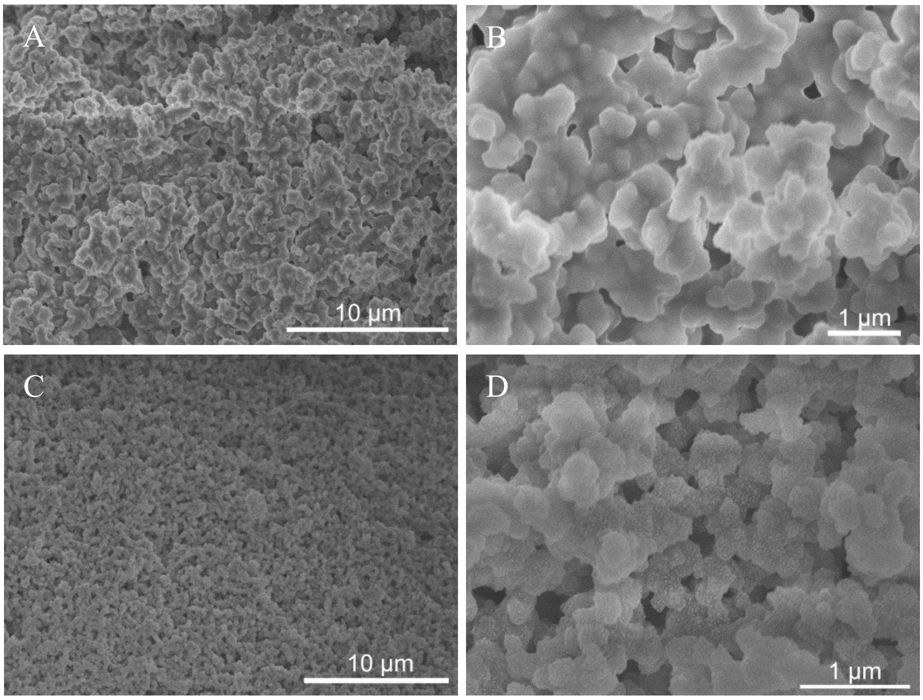


**Figure S15.** SEM images of the samples prepared with the initial mass composition of [$C_1$DVIM]Br 0.3g, [$C_4$MIM]Br 6 g, co-solvent 0.75 g and AIBN 0.03 g. Co-solvent: (A, B) EtOH; (C, D) DMSO.

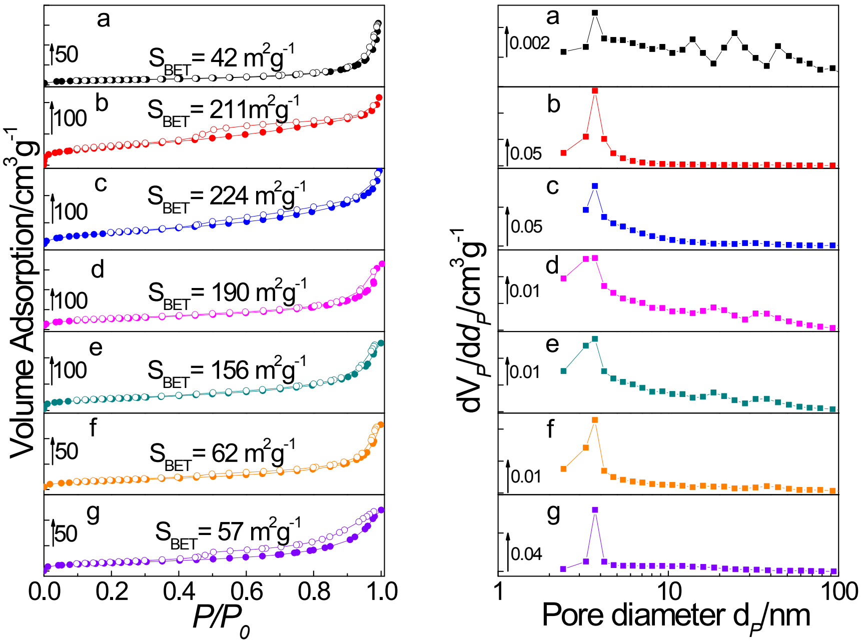


**Figure S16.** $N_2$ sorption isotherms (left) and the corresponding pore size distribution curves (right) of the samples with initial mass composition of [$C_1$DVIM]$BF_4$ 0.3g, [$C_4$MIM]$BF_4$ 6 g, co-solvent *x* g, and AIBN 0.03 g. $H_2O$ is used as the co-solvent for (a) *x*=0, (b) *x*= 0.25, (c) *x*= 0.5, (d) *x*= 0.75, (e) *x*= 1. (f) 0.5 g DMF or (g) DMSO is used as co-solvent.

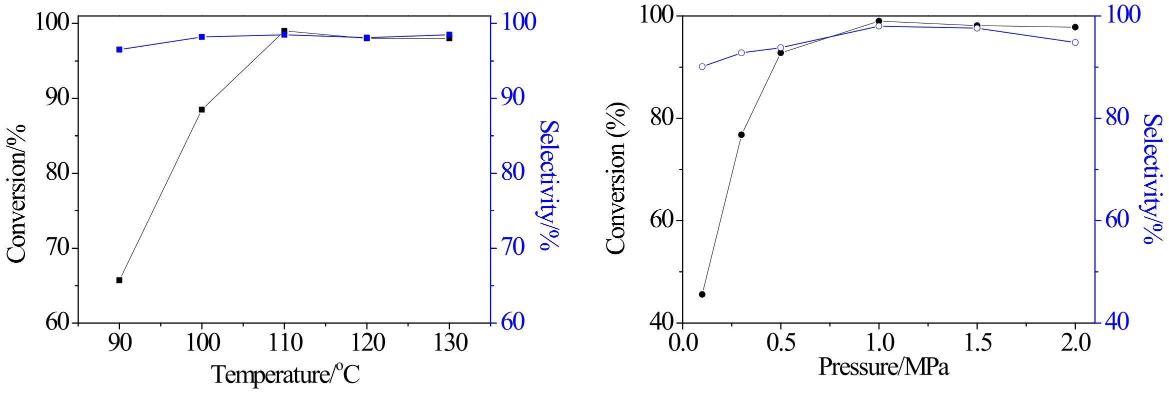


**Figure S17.** The effect of reaction temperature and pressure. Reaction conditions: SO (10 mmol), catalyst PDMBr (0.05 g) and reaction time (4 h).

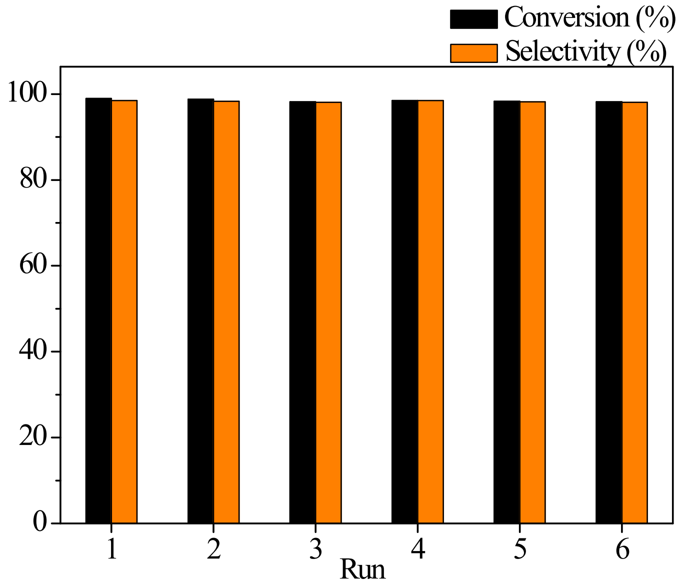


**Figure S18.** Catalytic reusability of PDMBr for cycloaddition of $CO_2$ to styrene oxide. Reaction conditions: SO 10 mmol, catalyst PDMBr 0.05 g (1.3 mol%), $CO_2$ pressure 1.0 MPa, 110 °C, 4 h.

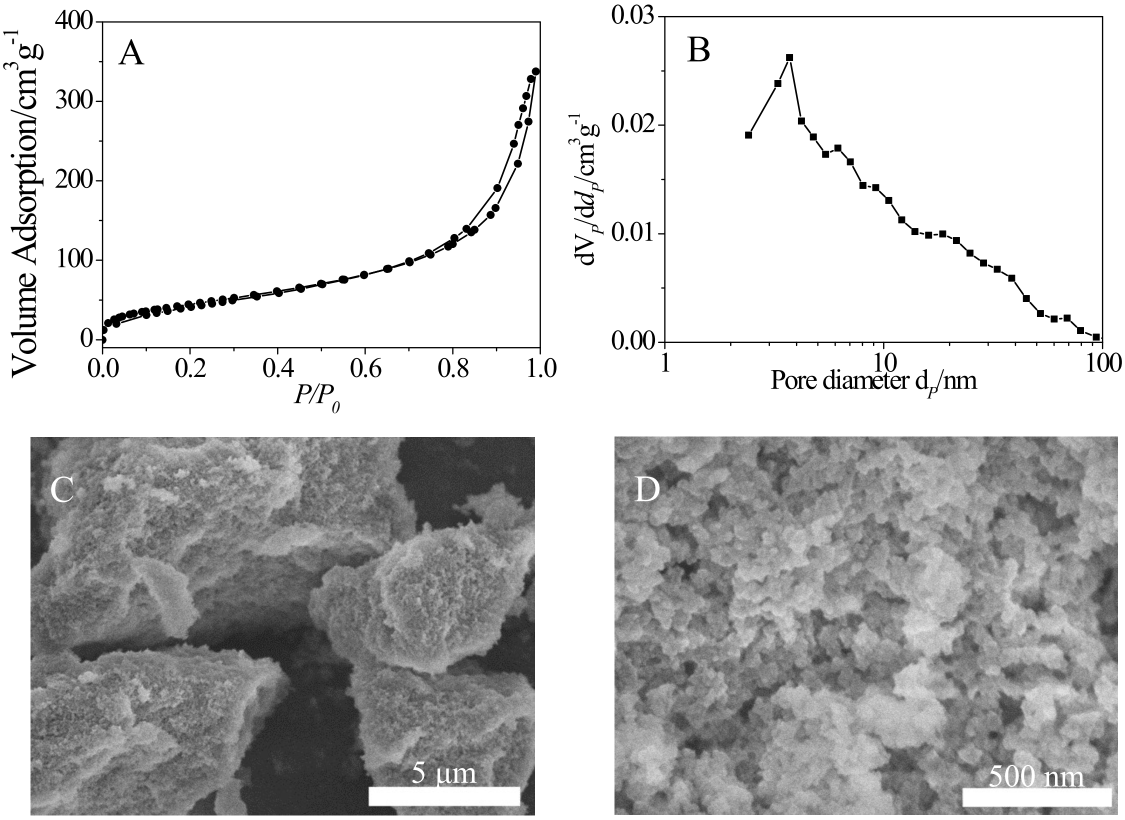


**Figure S19.** (A) $N_2$ sorption isotherm, (B) pore size distribution curve and (C, D) SEM images of the recovered catalyst PDMBr from cycloaddition of $CO_2$ to styrene oxide.

**Table S1.** Textural properties of the samples prepared with the initial mass composition of [$C_1$DVIM]Br 0.3 g, [$C_4$MIM]Br 6 g, $H_2O$ *x* g and AIBN 0.03 g.

| Entry | $H_2O$ (g) | $S_{BET}$[a] ($m^2$ $g^{-1}$) | $V_p$[b] ($cm^3$ $g^{-1}$) | $D_{av}$[c] (nm) |
|---|---|---|---|---|
| 1[d] | 0 | - | - | - |
| 2 | 0.25 | 2 | 0.01 | 14.3 |
| 3 | 0.5 | 147 | 0.32 | 8.7 |
| 4 | 0.75 | 205 | 0.57 | 11.0 |
| 5 | 1 | 189 | 0.51 | 10.6 |
| 6 | 1.5 | 170 | 0.44 | 10.3 |
| 7 | 2 | 62 | 0.14 | 9.2 |
| 8[e] | 0.75 | 197 | 0.59 | 12.1 |
| 9[f] | 0.75 | 12.0 | 0.08 | 26.0 |
| 10[g] | 0.75 | 11.5 | 0.06 | 21.6 |

[a] BET surface area. [b] Total pore volume. [c] Average pore diameter. [d] Undetectable. [e] Recovered [$C_4$MIM]Br is used as solvent. Initial mass composition: [$C_1$DVIM]Br 0.3g, [f] DMF or [g] DMSO 6 g, $H_2O$ 0.75 g and AIBN 0.03 g

**Table S2** The elemental analysis data of different samples.

| Samples | Elemental analysis Calcd | | | | Elemental analysis Found | | | |
|---|---|---|---|---|---|---|---|---|
| | C % | N % | H % | C/N | C % | N % | H % | C/N |
| [$C_1$DVIM]Br | 36.49 | 15.47 | 3.90 | 2.36 | 36.17 | 15.3 | 3.89 | 2.36 |
| [$C_1$DVIM]BF | 35.15 | 14.91 | 3.75 | 2.36 | 34.90 | 14.85 | 3.536 | 2.35 |
| PDMBr0.75 | 36.49 | 15.47 | 3.90 | 2.36 | 34.66 | 14.37 | 5.26 | 2.41 |
| PDMBr-E | 36.49 | 15.47 | 3.90 | 2.36 | 34.85 | 14.51 | 5.03 | 2.40 |
| PDMBr-H | 36.49 | 15.47 | 3.90 | 2.36 | 34.05 | 14.13 | 5.65 | 2.41 |
| PDMBF | 35.15 | 14.91 | 3.75 | 2.36 | 34.05 | 14.21 | 4.424 | 2.39 |

**Table S3** Textural properties of the samples prepared with the initial mass composition of imidazolium salt monomer 0.3 g, [$C_4$MIM]Br 6 g, $H_2O$ 0.75 g, and AIBN 0.03 g.

| Entry | Imidazolium salt monomer | $S_{BET}^a$ ($m^2$ $g^{-1}$) | $V_p^b$ ($cm^3$ $g^{-1}$) | $D_{av}^c$ (nm) |
|---|---|---|---|---|
| 1 | [$C_1$DVIM]Br | 205 | 0.57 | 11.0 |
| 2 | [$C_2$DVIM]Br | 45 | 0.18 | 15.6 |
| 3[d] | [$C_4$DVIM]Br | - | - | - |
| 4[d] | [$C_2$VIM]Br | - | - | - |

[a] BET surface area. [b] Total pore volume. [c] Average pore diameter. [d] Undetectable.

**Table S4** Textural properties of the samples prepared with the initial mass composition of [$C_1$DVIM]Br 0.3 g, IL 6 g, $H_2O$ 0.75 g, and AIBN 0.03 g.

| Entry | IL | $S_{BET}$[a] ($m^2\ g^{-1}$) | $V_p$[b] ($cm^3\ g^{-1}$) | $D_{av}$[c] (nm) |
|---|---|---|---|---|
| 1 | [$C_2$MIM]Br | 145 | 0.28 | 7.7 |
| 2 | [$C_4$MIM]Br | 205 | 0.57 | 11.0 |
| 3 | [$C_6$MIM]Br | 222 | 0.74 | 13.4 |
| 4[d] | [$C_8$MIM]Br | - | - | - |
| 5[d] | [$C_4$Py]Br | - | - | - |
| 6 | [$P_{4444}$]Br | 55 | 0.15 | 11.0 |
| 7 | TPABr | 260 | 0.62 | 9.6 |
| 8 | TBABr | 181 | 0.92 | 20.3 |

[a] BET surface area. [b] Total pore volume. [c] Average pore diameter. [d] Undetectable.

**Table S5** Textural properties of the samples prepared with the initial mass composition of [$C_1$DVIM]Br 0.3 g, [$C_4$MIM]Br *x* g, $H_2O$ 0.75 g and AIBN 0.03 g.

| Entry | [$C_4$MIM]Br (g) | $S_{BET}$[a] ($m^2\ g^{-1}$) | $V_p$[b] ($cm^3\ g^{-1}$) | $D_{av}$[c] (nm) |
|---|---|---|---|---|
| 1 | 0.5 | < 1 | < 0.01 | 11.0 |
| 2 | 1 | 165 | 0.53 | 12.7 |
| 3 | 2 | 171 | 0.39 | 9.1 |
| 4 | 3 | 219 | 0.47 | 8.5 |
| 5[d] | 9 | - | - | - |

[a] BET surface area. [b] Total pore volume. [c] Average pore diameter. [d] Undetectable.

**Table S6** Textural properties of the samples prepared with the initial mass composition of [$C_1$DVIM]Br 0.3 g, [$C_4$MIM]Br 6 g, cosolvent 0.75 g and AIBN 0.03 g.

| Entry | Co-solvent | $S_{BET}^{a}$ ($m^2$ $g^{-1}$) | $V_p^{b}$ ($cm^3$ $g^{-1}$) | $D_{av}^{c}$ (nm) |
|---|---|---|---|---|
| 1 | EtOH | 4 | 0.01 | 8.9 |
| 2 | AcOH | 10 | 0.05 | 17.5 |
| 3 | DMF | 7 | 0.03 | 16.7 |
| 4 | DMSO | 10 | 0.05 | 19.2 |

[a] BET surface area. [b] Total pore volume. [c] Average pore diameter.

**Table S7.** Textural properties of the samples prepared with the initial mass composition of [$C_1$DVIM]$BF_4$ 0.3 g, [$C_4$MIM]$BF_4$ 6 g, co-solvent *x* g and AIBN 0.03 g.

| Entry | Co-solvent/g | $S_{BET}^{a}$ ($m^2$ $g^{-1}$) | $V_p^{b}$ ($cm^3$ $g^{-1}$) | $D_{av}^{c}$ (nm) |
|---|---|---|---|---|
| 1 | $H_2O$/0 | 42 | 0.2 | 20 |
| 2 | $H_2O$/0.25 | 211 | 0.31 | 5.9 |
| 3 | $H_2O$/0.5 | 224 | 0.40 | 7.2 |
| 4 | $H_2O$/0.75 | 190 | 0.48 | 10.1 |
| 5 | $H_2O$/1 | 156 | 0.37 | 9.5 |
| 6 | DMF/0.5 | 62 | 0.17 | 11.2 |
| 7 | DMSO/0.5 | 57 | 0.18 | 12.2 |

[a] BET surface area. [b] Total pore volume. [c] Average pore diameter.

**Table S8.** Catalytic activity of different poly(ionic liquid)s for cycloaddition of $CO_2$ to styrene oxide.

| Entry | Catalyst | $CO_2$ (MPa) | Temp. (°C) | Time (h) | Yield (%) | Ref. |
|---|---|---|---|---|---|---|
| 1 | Imidazolium-based polymeric ionic liquids Poly[vbim]Cl | 5 | 140 | 24 | 67 | [7] |
| 2 | Copolymerization of phosphorous ionic liquid PPIL-co-PEDMA | 3 | 160 | 48 | 83.7 | [8] |
| 3 | Phosphonium chlorides immobilized on fluorous polymer (homogeneous catalyst) | 8 | 150 | 8 | 95 | [9] |
| 4 | Silicon-based poly-imidazolium salts | 1 | 110 | 2 | 96 | [10] |
| 5 | Fluoro-functionalized PILs | 1 | 120 | 9 | 96 | [11] |
| 6 | Hydroxyl-functionalized PILs copolymer | 2.5 | 130 | 6 | 90 | [12] |
| 7 | Cross-linked-polymer-supported IL PVBIMCl | 6 | 110 | 7 | 79.1 | [13] |
| 8 | Mesoporous zwitterionic poly(ionic liquid)s | 1 | 150 | 24 | 82 | [14] |
| 9 | Chitosan functionalized ionic liquid CS-EMImBr | 2 | 120 | 4 | 85 | [15] |
| 10 | MCM-41-Imi/Br | 3 | 100 | 4 | 97.7 | [16] |
| 11 | Polymers anchored with carboxyl-functionalized di-cation ionic liquids | 2.5 | 130 | 4 | 99.4 | [17] |
| 12 | Mesoporous polymer supported imidazolium-based ionic liquid FDU-HEIMBr | 1 | 110 | 3 | 95 | [18] |
| 13 | PDMBr | 1 | 110 | 4 | 96.8 | This work |

**Table S9.** Catalytic activity of MOF or microporous polymers for cycloaddition of $CO_2$ with epoxides under atmospheric pressure.

| Entry | Catalyst | Co-catalyst | Epoxide | Solvent | Temp. (°C) | Time (h) | Yield (%) | Ref. |
|---|---|---|---|---|---|---|---|---|
| 1 | PP-Br | - | 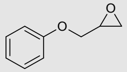 | DMF | 90 | 70 | 80.4 | [19] |
| 2 | MOF-5 | *n*-$Bu_4NBr$ | 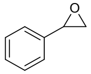 | - | 50 | 15 | 92 | [20] |
| 3 | Co-CMP100 | *n*-$Bu_4NBr$ | 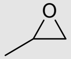 | - | 25 | 48 | 81.5 | [21] |
| 4 | 2D-CCB | n-$Bu_4NBr$ | 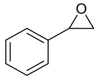 | - | 100 | 12 | 91.1 | [22] |
| 5 | Hf-NU-1000 | *n*-$Bu_4NBr$ | 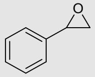 | - | 25 | 56 | 100 | [23] |
| 6 | [SnIV(TNH$_2$PP)(OTf)$_2$]/CM-MIL-101 | *n*-$Bu_4PBr$ | 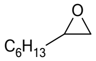 | DMF | 50 | 11 | 100 | [24] |
| 7 | USTC-253-TFA | *n*-$Bu_4NBr$ | 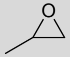 | - | 25 | 72 | 81.3 | [25] |
| 8 | BIT-C | *n*-$Bu_4NBr$ | 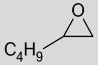 | - | 35 | 24 | 91 | [26] |
| 9 | | | 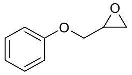 | | 70 | 48 | 90.0 | |
| 10 | PDMBr | - | 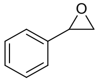 | - | 120 | 12 | 80.1 | This work |
| 11 | | | 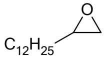 | | 120 | 48 | 97.1 | |

**Additional explanation for Tables S8 and S9 and activity comparison**

The comparison of catalytic activities is difficult due to the variation of the reaction conditions (substrate, reaction temperature, $CO_2$ pressure, co-catalyst, solvent, etc.) for different catalysts. The comparison of the activity under the atmospheric pressure condition is especially difficult because only several reports are related to the catalytic performances of heterogeneous catalysts at such a condition (Table S9). Up to now, there is no report of a heterogeneous catalyst that is able to promote the cycloaddition of $CO_2$ to epoxides under ambient conditions without a solvent or addition of an external homogeneous co-catalyst. In this work, PDMBr is the first metal-solvent-additive free recyclable heterogeneous catalyst for cycloaddition of $CO_2$ under ambient conditions.

Table S9 lists the previous heterogeneous catalysts for cycloaddition of $CO_2$ under different conditions. Quaternary phosphonium-containing microporous polymer (PP-Br) gave the yield of 80.4% (90 °C, 70 h) for the substrate of glycidyl phenyl ether when N,N-dimethylformamide (DMF) was used as the solvent (Table S9, entry 1). By contrast, our synthesized PDMBr catalyst exhibits the higher yield (90%) for the same substrate at the lower temperature (70 °C) and shorter time (48 h) under the solvent-free condition, obviously demonstrating much higher activity than PP-Br. In addition, metal-organic frameworks (MOFs) or metal-coordinated conjugated microporous polymers (CMPs) in previous reports displayed efficient catalytic activities for cycloaddition of styrene oxide or propylene epoxide with $CO_2$ into cyclic carbonates (1 atm, 25-100 °C) (Table S9, entry 2-6), but the homogeneous co-catalysts like *n*-$Bu_4NBr$ or *n*-$Bu_4PBr$ were needed to get those excellent results; otherwise, they presented inferior catalytic performance even at relatively high temperature and $CO_2$ pressure.[27-30] In other words, those catalysts are short of specific basic sites and rely on the acidity of the metal cores within the framework and external nucleophiles;[31,32] therefore, they themselves are unable to efficiently catalyze the ambient cycloaddition of $CO_2$ to epoxides. The above comparison allows drawing that PDMBr of this work is a more efficient heterogeneous catalyst for cycloaddition of $CO_2$ to epoxides under ambient conditions.

For further insight into the present catalyst PDMBr, it is also evaluated under the relative mild conditions of 110 °C and 1 MPa, according to very recent reports.[10,18,32-35] Under such conditions, some previous heterogeneous catalysts presented high activities, nonetheless, low yield was observed over the inert substrates like aliphatic long carbon-chain alkyl epoxides,[10,15] and decreased activity still occurred in recycling tests.[10,18,19] Generally, styrene oxide is more reactive than long carbon-chain alkyl epoxides, but less reactive than those robust substrates such as glycidyl ethers and epichlorohydrin. In the early studies (Table S8, entry 1-3, 6, 7-11), the conversion of styrene oxide usually took place at much higher temperature and $CO_2$ pressure with long reaction time, whereas our PDMBr is more active, giving a conversion of 99.0% and selectivity of 98.5% at milder conditions. Compared with recently reported ones (Table S8, entry 4, 5, 12), PDMBr also exhibits better or at least comparable activity, implying that PDMBr is among the most efficient IL-related heterogeneous catalysts for this reaction. Further, other than styrene oxide, PDMBr is able to convert both the robust and inert substrates to corresponding cyclic carbonates with high yields under the relative mild conditions of 110 °C and 1 MPa (Table 2), which has never been achieved before. An additional advantage of PDMBr is its highly stable activity revealed by the recycling test.

Based on above analysis, our obtained material PDMBr is an efficient catalyst for $CO_2$ conversion. It is the most efficient heterogeneous catalyst under ambient conditions considering that the high yields are achieved for various substrates without aided by any metals, additives, and solvents.

## References for Supporting Information


[1] H. Chu, C. Yu, Y. Wan, D. Zhao, *J. Mater. Chem.*, 2009, **19**, 8610-8618.
[2] F. Liu, W. Li, Q. Sun, L. Zhu, X. Meng, Y.-H. Guo, F.-S. Xiao, *ChemSusChem*, 2011, **4**, 1059-1062.
[3] G. Liu, M. Hou, J. Song, T. Jiang, H. Fan, Z. Zhang, B. Han, *Green Chem.*, 2010, **12**, 65-69.
[4] L. Peng , J. Zhang, S. Yang , B. Han , X. Sang , C. Liu, G. Yang, *Chem. Commun.*, 2014, **50**, 11957-11960.
[5] H. Zhao, L. Li, Y. Wang, R. Wang, *Sci. Rep.*, 2014, **4**, 5478.

[6] J. Xu , F. Wu , Q. Jiang,Y.-X. Li, *Catal. Sci. Technol.*, 2015, **5**, 447-454.
[7] S. Ghazali-Esfahani, H. Song, E. Păunescu, F. D. Bobbink, H. Liu, Z. Fei, G. Laurenczy, M. Bagherzadeh, N. Yana, P. J. Dyson, *Green Chem.*, 2013, **15**, 1584-1589.
[8] Y. Xiong, H. Wang, R. Wang, Y. Yan, B. Zheng, Y. Wang, *Chem. Commun.*, 2010, **46**, 3399-3401.
[9] Q.-W. Song, L.-N. He, J.-Q. Wang, H. Yasuda, T. Sakakura, *Green Chem.*, 2013, **15**, 110-115.
[10] J. Wang, J. Leong and Y. Zhang, *Green Chem*., 2014, **16**, 4515-4519.
[11] Z.-Z. Yang, Y. Zhao, G. Ji, H. Zhang, B. Yu, X. Gao, Z. Liu, *Green Chem*., 2014, **16**, 3724-3728.
[12] T.-Y. Shi, J.-Q. Wang, J. Sun, M.-H. Wang, W.-G. Cheng, S.-J. Zhang, *RSC Adv.*, 2013, **3**, 3726-3732.
[13] Y. Xie, Z. Zhang, T. Jiang, J. He, B. Han, T. Wu, K. Ding, *Angew. Chem. Int. Ed.*, 2007, **46**, 7255-7258.
[14] S. Soll, P. Zhang, Q. Zhao, Y. Wang, J. Yuan, *Polym. Chem.*, 2013, **4**, 5048-5051.
[15] J. Sun, J. Wang, W. Cheng, J. Zhang, X. Li, S. Zhang, Y. She, *Green Chem.*, 2012, **14**, 654-660.
[16] J. N. Appaturi, F. Adam, *Appl. Catal. B*, 2013, **136-137**, 150-159.
[17] W.-L. Dai, B. Jin, S.-L. Luo, X.-B. Luo, X.-M. Tu, C.-T. Au, *Catal. Sci. Technol.*, 2014, **4**, 556-562.
[18] W. Zhang, Q. Wang, H. Wu, P. Wu, M. He, *Green Chem.*, 2014, **16**, 4767-4774.
[19] Q. Zhang, S. Zhang, S. Li, *Macromolecules*, 2012, **45**, 2981-2988.
[20] J. Song, Z. Zhang, S. Hu, T. Wu, T. Jiang, B. Han, *Green Chem.*, 2009, **11**, 1031-1036.
[21] Y. Xie, T.-T. Wang, X.-H. Liu, K. Zou, W.-Q. Deng, *Nat. Commun*., 2014, **4**, 1960.
[22] A. C. Kathalikkattil, R. Roshan, J. Tharun, H.-G. Soek, H.-S. Ryu, D.-W. Park, *ChemCatChem*, 2014, **6**, 284-292.
[23] M. H. Beyzavi, R. C. Klet, S. Tussupbayev, J. Borycz, N. A. Vermeulen, C. J. Cramer, J. F. Stoddart, J. T. Hupp, O. K. Farha, *J. Am. Chem. Soc.*, 2014, **136**, 15861-15864.
[24] F. Zadehahmadi, F. Ahmadi, S. Tangestaninejad, M. Moghadam, V. Mirkhani, I. Mohammadpoor-Baltork, R. Kardanpour, *J. Mol. Catal. A*, 2015, **398**, 1-10.
[25] Z.-R. Jiang, H. Wang, Y. Hu, J. Lu, H.-L. Jiang, *ChemSusChem*, 2015, **8**, 878-885.
[26] B. Zou, L. Hao , L.-Y. Fan, Z.-M. Gao, S.-L. Chen, H. Li, C.-W. Hu, *J. Catal.*, 2015, **329**, 119-129.
[27] T. Lescouet, C. Chizallet, D. Farrusseng, *ChemCatChem*, 2012, **4**, 1725-1728.
[28] H.-Y. Cho, D.-A. Yang, J. Kim, S.-Y. Jeong, W.-S. Ahn, *Catal. Today*, 2012, **185**, 35-40.
[29] D.-A. Yang, H.-Y. Cho, J. Kim, S.-T. Yang, W.-S. Ahn, *Energy Environ. Sci.*, 2012, **5**, 6465-6473.
[30] J. Kim, S.-N. Kim, H.-G. Jang, G. Seo, W.-S. Ahn, *Appl. Catal. A*, 2013, **453**, 175-180.
[31] V. D'Elia, J. D. A. Pelletier, J.-M. Basset, *ChemCatChem*, 2015, **7**, 1906-1917.
[32] Y. Zhang,D. S. W. Lim, DOI: 10.1002/cssc.201500745.
[33] W. Zhang, T. Liu, H. Wu, P. Wu, M. He, *Chem. Commun.*, 2015, **51**, 682-684.
[34] Q. He, J. W. O'Brien, K. A. Kitselman, L. E. Tompkins, G. C. T. Curtis, F. M. Kerton, *Catal. Sci. Technol.*, **2014**, 4, 1513-1528.
[35] T. Ema, K. Fukuhara, T. Sakai, M. Ohbo, F.-Q. Bai, J.-y. Hasegawa, *Catal. Sci. Technol.*, **2015**, 5, 2314-2321.
